# Supplementary material for: Raised inflammatory markers as a predictor of one-year mortality: a cohort study in primary care in the UK using electronic health record data
Source: BMJ Open. 2020 Oct 15;10(10):e036027. doi: 10.1136/bmjopen-2019-036027 (PMC7566728; doi:10.1136/bmjopen-2019-036027)
Supplement: Supplementary data [file bmjopen-2019-036027supp001.pdf]

Supplementary Table 1: Characteristics of the tested and untested cohort, compared to the UK population

Patient Characteristic	Tested Cohort	Untested controls	National Population	GP Attendance
**Gender:**	**(159,325)**	**(39,928)**	*	**
Male	38.0%	38.2%	48.7%	39.8%
Female	62.0%	61.8%	51.3%	60.2%
**Age Group:**	**(159,325)**	**(39,928)**	*	**
18-29	10.9%	10.8%	20.2%	12.5%
30-39	11.4%	11.5%	16.4%	13.7%
40-49	15.9%	15.8%	17.8%	14.7%
50-59	17.6%	17.7%	16.3%	14.1%
60-69	17.7%	17.3%	13.9%	16.3%
70-79	15.1%	15.4%	9.4%	15.5%
≥80	11.5%	11.4%	6.0%	13.2%
**IMD Socioeconomic Status***:**	**(87,839)**	**(22,062)**		
1 (least deprived)	23.1%	23.9%	20.0%	
2	21.9%	22.2%	20.0%	
3	21.6%	21.4%	20.0%	
4	19.1%	19.0%	20.0%	
5 (most deprived)	14.2%	13.6%	20.0%	
**Ethnicity***:**	**(75,802)**	**(17,868)**	**	
White	87.8%	87.7%	85.4%	
Non-white	12.3%	12.4%	14.6%	

* Based on UK population estimates in mid-2014[1]
** Based on ONS – National Consultation Rates Report (2008)[2] (age 20-30 not 18 – 30)
*** IMD linkage and ethnicity data was not available for the complete dataset, hence a lower denominator for these figures shown

[1] Office for National Statistics. *Population Estimates for UK, England and Wales, Scotland and Northern Ireland, Mid-2014*. (2014).

[2] Hippisley-Cox, J. *ONS Consultations Report 20 - QRESEARCH calendar year consultations 2008*. (2008).

Supplementary table 2: Cause of death amongst patients with one or more raised inflammatory markers at the index date subdivided by age category (n=1,872 deaths)

Age group	Cause of death n (%)			
	Cancer	Cardiovascular	Respiratory	Other
<30	0 (0)	0 (0)	0 (0)	1 (100)
30-39	4 (40.0)	1 (10.0)	0 (0)	5 (50.0)
40-49	21 (45.7)	4 (8.7)	1 (2.2)	20 (43.5)
50-59	56 (56.6)	16 (16.2)	9 (9.1)	18 (18.2)
60-69	152 (61.0)	31 (12.5)	25 (10.0)	41 (16.5)
70-79	199 (46.6)	88 (20.6)	59 (13.8)	81 (19.0)
≥80	264 (25.4)	309 (29.7)	170 (16.4)	297 (28.6)
Total	696 (37.2)	449 (24.0)	264 (14.1)	463 (24.7)
