# Supplementary material for: Raised inflammatory markers as a predictor of one-year mortality: a cohort study in primary care in the UK using electronic health record data
Source: BMJ Open. 2020 Oct 15;10(10):e036027. doi: 10.1136/bmjopen-2019-036027 (PMC7566728; doi:10.1136/bmjopen-2019-036027)
Supplement: Author's manuscript [file bmjopen-2019-036027.draft_revisions.pdf]

BMJ Open is committed to open peer review. As part of this commitment we make the peer review history of every article we publish publicly available.

When an article is published we post the peer reviewers' comments and the authors' responses online. We also post the versions of the paper that were used during peer review. These are the versions that the peer review comments apply to.

The versions of the paper that follow are the versions that were submitted during the peer review process. They are not the versions of record or the final published versions. They should not be cited or distributed as the published version of this manuscript.

BMJ Open is an open access journal and the full, final, typeset and author-corrected version of record of the manuscript is available on our site with no access controls, subscription charges or pay-per-view fees (http://bmjopen.bmj.com).

If you have any questions on BMJ Open's open peer review process please email info.bmjopen@bmj.com

BMJ Open

Raised inflammatory markers in primary care as a predictor of one-year mortality: A cohort study using CPRD

Journal:	*BMJ Open*
Manuscript ID	bmjopen-2019-036027
Article Type:	Original research
Date Submitted by the Author:	26-Nov-2019
Complete List of Authors:	Watson, Jessica; University of Bristol, Centre for Academic Primary Care<br>Whiting, Penny; University of Bristol, School of Social and Community Medicine<br>Salisbury, Chris; University of Bristol, Academic Unit of Primary Health Care<br>Banks, Jonathan; University of Bristol, Social & Community Medicine<br>Hamilton, Willie; University of Exeter Medical School, Primary Care Diagnostics
Keywords:	PRIMARY CARE, EPIDEMIOLOGY, Chemical pathology < PATHOLOGY, GERIATRIC MEDICINE

SCHOLARONE™
Manuscripts

I, the Submitting Author has the right to grant and does grant on behalf of all authors of the Work (as defined in the below author licence), an exclusive licence and/or a non-exclusive licence for contributions from authors who are: i) UK Crown employees; ii) where BMJ has agreed a CC-BY licence shall apply, and/or iii) in accordance with the terms applicable for US Federal Government officers or employees acting as part of their official duties; on a worldwide, perpetual, irrevocable, royalty-free basis to BMJ Publishing Group Ltd ("BMJ") its licensees and where the relevant Journal is co-owned by BMJ to the co-owners of the Journal, to publish the Work in this journal and any other BMJ products and to exploit all rights, as set out in our licence.

The Submitting Author accepts and understands that any supply made under these terms is made by BMJ to the Submitting Author unless you are acting as an employee on behalf of your employer or a postgraduate student of an affiliated institution which is paying any applicable article publishing charge ("APC") for Open Access articles. Where the Submitting Author wishes to make the Work available on an Open Access basis (and intends to pay the relevant APC), the terms of reuse of such Open Access shall be governed by a Creative Commons licence – details of these licences and which Creative Commons licence will apply to this Work are set out in our licence referred to above.

Other than as permitted in any relevant BMJ Author's Self Archiving Policies, I confirm this Work has not been accepted for publication elsewhere, is not being considered for publication elsewhere and does not duplicate material already published. I confirm all authors consent to publication of this Work and authorise the granting of this licence.

For peer review only

Raised inflammatory markers in primary care as a predictor of one-year mortality: A cohort study using CPRD

Jessica Watson[1,2], Penny Whiting[1,2], Chris Salisbury[1,2], Jonathan Banks[1,2], Willie Hamilton[3]

1. Population Health Sciences, Bristol Medical School, University of Bristol, Bristol, UK
2. NIHR CLAHRC West, Bristol, UK
3. University of Exeter Medical School, Exeter, UK

Corresponding author:

Dr Jessica Watson

Population Health Sciences

Canynge Hall

39 Whatley Road

Bristol, BS8 2PS

Jessica.Watson@bristol.ac.uk

Tel: (+44) 07837002664

Word count: 2,094 (excluding abstract, references, figures and tables)

For peer review only

Abstract

Objectives

The aim of this study was to examine one-year all-cause mortality in a cohort of primary care patients in whom inflammatory markers including C-reactive protein (CRP), erythrocyte sedimentation rate (ESR) and plasma viscosity (PV), had been tested.

Design

Observational cohort study using GP Electronic Health Records from the Clinical Practice Research Datalink, with linkage to ONS Death Registry.

Setting

UK Primary Care

Participants

159,325 patients with inflammatory marker tests done in 2014 and 39,928 age, sex and practice matched controls without inflammatory marker testing. ONS Death registry data was available for 109,966 participants.

Primary and secondary outcome measures

One-year mortality in those with raised inflammatory markers compared to normal inflammatory markers and untested controls. Sub-analyses stratified one-year mortality by age-group, gender and cause of death.

For peer review only

Results

Patients with a raised inflammatory marker (n=47,797) had an overall one-year all-cause mortality of 6.89%, compared to 1.41% in those with normal inflammatory markers (p<0.001) and 1.62% in untested controls. A raised CRP is associated with the highest mortality rate at 8.76% compared to 4.99% for ESR and 4.66% for PV. One-year mortality is higher in men with a raised inflammatory marker compared to women (9.78% versus 5.29%). The c-statistic of a simple mortality prediction model containing age, sex and CRP test result is 0.89.

Conclusions

Inflammatory markers are a strong predictor of all-cause mortality in primary care, with a c-statistic comparable to several previously developed frailty indices. Future research should consider the added value of CRP testing, in combination with other risk factors, to improve prediction of mortality in primary care. Evidence based interventions for frailty are needed alongside predictive tools.

For peer review only

Strengths and limitations of this study

- Using a large observational primary care electronic health records dataset we demonstrate a strong association between raised inflammatory markers and one-year mortality
- As test results were transferred electronically to the GP record, there is a very low risk of transcription error or bias.
- Use of ONS death registry data increased the accuracy of recording of mortality, the primary outcome.
- Previous studies have focussed on predictors of mortality in the elderly, yet we were able to study mortality across all age groups.
- Future research should consider the added value of CRP testing in combination with other risk factors, to improve prediction of mortality in primary care

For peer review only

Background

Identification of patients at increased risk of mortality is important in the context of increasing multimorbidity and an ageing population, with the aim of helping facilitate the planning and delivery of services. Multiple risk tools have therefore been developed to predict mortality; these can be used to help predict frailty, unplanned hospital admissions and to allow targeted interventions to people at an increased mortality risk. The National Institute for Health and Care Excellence (NICE) multimorbidity guidelines systematically reviewed 41 of these mortality risk tools; the majority were of low or very low quality and a need for further research in this area was identified.[1] Current risk tools include variables such as disease status, sociodemographic factors and laboratory test results (eg anaemia, raised platelets): however, none in current use include an inflammatory marker test.

Inflammatory markers such as C reactive protein (CRP), erythrocyte sedimentation rate (ESR) and plasma viscosity (PV) are commonly used in primary care to aid diagnosis and monitoring of infections and inflammatory conditions. Cohort studies in the general population have reported inflammatory markers as predicting future mortality,[2-4] particularly from cardiovascular disease.[5] CRP has also been shown to have predictive value for mortality in hospital settings.[6-8] The clinical relevance of these findings in primary care settings, and over the shorter term, has not previously been described.

The aim of this study was to examine one-year all-cause mortality in a cohort of primary care patients in whom inflammatory marker bloods had been tested.

Methods

This was a cohort study of 160,000 patients aged >18 from the Clinical Practice Research Datalink (CPRD) with inflammatory marker blood testing in 2014, and 40,000 age, sex and practice matched

For peer review only

controls without inflammatory marker testing also in 2014. The methods have been described fully previously.[9] Patients were excluded from the analysis if the inflammatory marker test result was missing (n=673) or if results were so abnormal as to be considered spurious (n=2). Linkage to ONS death registry data was available for 109,966. The three inflammatory marker tests studied were C-reactive protein (CRP), erythrocyte sedimentation rate (ESR) and plasma viscosity (PV). We defined a raised inflammatory marker using the mean upper limit of normal laboratories within our study. For CRP this was 6.8mg/l, for simplicity rounded to 7mg/l; for PV 1.72mPa.s. For ESR this was stratified by gender and age.[9] When the same inflammatory marker was repeated on the same day (n=231), we retained the highest value. The index date was defined as the first date of inflammatory marker testing in 2014, with one-year mortality defined as death within one year of this index date. Date of death was defined as the earlier date of recorded death in either CPRD or ONS death registry. Cause of death was available from death certification data in 3,141 out of 5,512 deaths where ONS linkage was available.

Analysis

The primary analysis compared one-year mortality in those with raised versus normal inflammatory markers and compared to untested controls. Sub-analyses stratified one-year mortality by age group and gender and cause of death. For each of the three tests (CRP, ESR and PV), dichotomised test results were cross-classified with the reference standard one-year mortality, allowing sensitivity and specificity to be calculated. Logistic regression was used to calculate diagnostic odds ratios, with and without adjustment for age and gender.

Test results were also analysed on a continuous scale, using logistic regression to determine the dose response relationship between inflammatory marker test result and mortality. The area under receiver operator curve (AUC), otherwise known as the c-statistic, was calculated using a logistic regression model using test result on a continuous scale, with log transformation due to the skewed

For peer review only

nature of the data, with and without age and gender as covariates. Comparisons of the c-statistic were made using DeLong method.[10] All analyses were done using Stata15.[11]

Results

After exclusions there were 159,325 tested patients of whom 114,198 (71.7%) had a CRP test, 92,325 (58.0%) an ESR test, and 15,994 (10%) a PV test; 47,797 (30%) had one or more raised inflammatory marker on the index date. In total 5,512 patients died within one-year of the index date; 648 deaths in the untested group, 1,572 deaths in the normal inflammatory marker group and 3,292 deaths in the group with one or more raised inflammatory marker.

Overall mortality rates (Table 1)

Patients with a raised inflammatory marker (n=47,797) had an overall one-year mortality of 6.89%, compared to 1.41% in those with normal inflammatory markers (p<0.001). In the untested comparison cohort, one-year mortality was 1.62%. The association between raised inflammatory markers and one-year mortality was seen in all age groups apart from the under 30-year olds. In older age groups the absolute increase in risk was considerable; a raised inflammatory marker in the over 80s was associated with a one-year mortality of 21.8%, compared to 8.6% in the over 80s with normal inflammatory markers.

Men with a raised inflammatory marker had a significantly higher one-year mortality rate than women with a raised inflammatory marker (9.78% versus 5.29%). Patients with a raised CRP had a one-year mortality of 8.76% compared to 4.99% for those with raised ESR and 4.66% for raised PV.

For peer review only

Table 1: One-year mortality (%, 95% CI) subdivided by age, gender and test result

	Untested (n=39,928)	Normal inflammatory markers (n=111,528)	Any raised inflammatory marker (n=47,797)	Raised CRP (n=29,164)	Raised ESR (n=23,138)	Raised PV (n=4,568)
Overall (n=199,253)	1.62 (1.50-1.75)	1.41 (1.34-1.48)	6.89 (6.66-7.11)	8.76 (8.43-9.08)	4.99 (4.71-5.27)	4.66 (4.05-5.27)
Age						
<30 (n=21,732)	0.09 (0.02-0.18)	0.04 (0.01-0.08)	0.08 (0.00-0.17)	0.13 (0.00-0.27)	0.00 (0.00-0.00)	0.0 (0.00-0.00)
30-39 (n=22,718)	0.04 (0.00-0.10)	0.08 (0.03-0.13)	0.37 (0.19-5.47)	0.50 (0.23-0.77)	0.28 (0.06-0.51)	0.0 (0.00-0.00)
40-49 (n=31,588)	0.19 (0.08-0.30)	0.15 (0.09-0.20)	1.12 (0.86-1.39)	1.54 (1.14-1.95)	0.92 (0.59-1.25)	0.90 (0.01-1.69)
50-59 (n=35,044)	0.41 (0.26-0.56)	0.41 (0.32-0.49)	2.37 (2.03-2.70)	2.98 (2.48-3.47)	1.79 (1.38-2.21)	1.62 (0.75-2.50)
60-69 (n=35,094)	0.84 (0.62-1.05)	0.91 (0.78-1.05)	4.96 (4.51-5.40)	6.72 (6.05-7.39)	3.88 (3.33-4.43)	3.27 (2.12-4.42)
70-79 (30,251)	2.27 (1.90-2.64)	2.51 (2.26-2.76)	9.39 (8.77-10.0)	11.38 (10.5-12.2)	7.35 (6.54-8.16)	6.32 (4.75-7.88)
>80 (22,826)	8.88 (8.05-9.70)	8.61 (8.07-9.16)	21.8 (20.9-22.7)	25.9 (24.7-27.1)	16.1 (14.9-17.3)	15.6 (12.9-18.3)
Gender						
Male (n=75,787)	1.86 (1.64-2.07)	1.58 (1.46-1.70)	9.78 (9.33-10.2)	11.48 (10.9-12.1)	7.98 (7.35-8.61)	6.63 (5.44-7.81)
Female (n=123,466)	1.48 (1.33-1.63)	1.30 (1.22-1.39)	5.29 (5.04-5.54)	6.99 (6.61-7.36)	3.65 3.36-3.94)	3.51 (2.84-4.18)

Table 2 shows the performance characteristics of inflammatory markers, including sensitivity, specificity and c-statistic. CRP had the highest sensitivity of the three tests at 67.8% and the greatest c-statistic at 0.78. Odds ratios (OR) reduced after adjustment for age and gender but were still significant with an adjusted OR for a raised CRP of 4.5 ($p<0.001$), 2.9 for raised ESR and 2.1 for raised PV.

A logistic regression model containing age (as a continuous variable) and gender had a c-statistic of 0.85, compared to 0.89 for a full model containing age, gender and CRP test result as a continuous variable ($p < 0.001$); 0.88 with age, gender and ESR ($p < 0.001$); and 0.87 with age gender and PV ($p < 0.001$).

For peer review only

Table 2: Performance characteristics of CRP, ESR and PV for predicting one-year mortality

				**Univariable logistic regression**		**Adjusted for age and gender**	
	Sensitivity	Specificity	C-statistic*	OR	P	OR	P
**CRP**	67.8% (66.3-69.3)	75.9% (75.6-76.2)	0.78 (0.77-0.78)	6.6 (6.2-7.1)	<0.001	4.5 (4.2-4.8)	<0.001
**ESR**	56.6% (54.4-58.7)	75.7% (75.4-75.9)	0.66 (0.65-0.67	4.1 (3.7-4.4)	<0.001	2.9 (2.7-3.2)	<0.001
**PV**	52.0% (47.0-56.9)	72.1% (71.3-72.8)	0.62 (0.60-0.64)	2.8 (2.3-3.4)	<0.001	2.1 (1.7-2.6)	<0.001

*C-statistic calculated using log transformed test results as a continuous variable

For peer review only

Repeat testing

Figure 1 shows the one-year mortality in patients according to the subsequent repeat inflammatory marker results, using the most common test performed; CRP. The fact that a CRP test was requested by a GP was in itself, predictive of increased mortality, with one-year mortality of 3.3% in the tested versus 1.6% in the untested cohort. This increased to 8.76% one-year mortality if a single CRP test was raised, 9.13% if a second test was persistently raised and 14.5% the second test was raised further still. Those with a raised inflammatory marker which was not subsequently rechecked had a one-year mortality rate of 10.2%, compared to 3.25% if a subsequent CRP normalised.

Figure 1: Flowchart of one-year mortality (95% confidence intervals) according to CRP test results. The right-hand column shows one-year mortality according to repeat test result; defined as the first CRP test performed in the 3 months following the index date

Dose response relationship

A dose response relationship was found between CRP result as a continuous variable and one-year mortality (figure 2). In 2,184 people with a CRP >=100mg/L overall one-year mortality was 20.2%. Similar associations, with wider confidence intervals, were found for ESR and PV (not shown).

Figure 2: Polynomial logistic regression of mortality against CRP test result as a continuous variable

Cause of death

Cause of death from ONS death certification was available for 3,141 out of 5,512 total deaths in the cohort. **Table 3** summarises the cause of death amongst patients with raised inflammatory markers, compared to those with normal inflammatory markers, and untested controls. The commonest cause of death in the 26,507 patients with raised inflammatory markers was cancer (696 deaths) followed by cardiovascular disease (449 deaths). Odds of mortality in the raised versus normal inflammatory marker groups was highest for cancer (adjusted OR 6.34), followed by infections (adjusted OR 4.11). However significant increased odds of mortality was seen for all disease categories with the exception of deaths due to falls, musculoskeletal causes and senility.

For peer review only

Table 3: Cause of death amongst patients with ONS death registry linkage (n=109,966)

Cause of death	Untested controls (n=22,069)		Normal inflammatory markers (n=61,390)		Raised inflammatory markers (n=26,507)		Comparison between normal and raised inflammatory markers	
	Number of deaths	1 year mortality (%)	Number of deaths	1 year mortality (%)	Number of deaths	1 year mortality (%)	Unadjusted odds ratio (CI)	Odds ratio, adjusted for age and gender (CI)
All-cause mortality	380	1.74	889	1.45	1,872	7.08	5.16* (4.76-5.60)	3.66* (3.37-3.99)
Cancer	86	0.39	195	0.32	696	2.63	8.46* (7.21-9.93)	6.34* (5.40-7.46)
Cardiovascular disease	115	0.52	295	0.48	449	1.69	3.57* (3.08-4.14)	2.18* (1.87- 2.54)
Respiratory	53	0.24	141	0.23	264	1.00	4.37* (3.56-5.36)	2.68* (2.18-3.30)
Dementia	38	0.17	69	0.11	119	0.45	4.01* (2.98-5.39)	2.21* (1.64-2.99)
Gastrointestinal	16	0.07	42	0.07	86	0.32	4.75* (3.29-6.88)	3.58* (2.46-5.20)
Genitourinary	8	0.04	16	0.03	39	0.15	5.65* (3.16-10.1)	3.13* (1.74-5.64)
Infection	5	0.02	11	0.02	29	0.11	6.11* (3.05-12.2)	4.08* (2.03-8.23)
Blood disorder	2	0.01	11	0.02	25	0.09	5.27* (2.59-10.7)	3.11 (1.52-6.37)
Senility	12	0.05	8	0.01	21	0.08	6.08* (2.69-13.7)	2.80 (1.23-6.38)
Musculoskeletal	3	0.01	8	0.01	14	0.05	4.05* (1.70-9.67)	2.40 (1.00-5.77)
Falls	4	0.02	10	0.02	6	0.02	1.39 (0.51-3.82)	0.80 (0.29-2.22)
Other	38	0.17	83	0.14	124	0.47	3.47* (2.62-4.59)	2.41* (1.82-3.20)

*p<0.001

Discussion

Inflammatory markers are a strong predictor of all-cause mortality in primary care. The association between raised inflammatory markers and all-cause mortality is seen in all age groups except the under thirties, with higher one-year mortality in men with raised inflammatory markers than women (9.78% vs 5.29%). Of the three tests examined, CRP has the highest predictive accuracy for mortality. The overall c-statistic of a model containing age, sex and CRP test result of 0.89 is comparable to

For peer review only

several previously developed frailty indices. Inflammatory markers could potentially be a simple indicator to improve prediction of life-expectancy in primary care.

Strengths and limitations

The major strength of this study is its size and its setting in primary care, making results relevant to clinical practice. As test results were transferred electronically to the GP record, there is a very low risk of transcription error or bias. Use of ONS death registry data increased the accuracy of recording of mortality, the primary outcome. Previous studies have focussed on predictors of mortality in the elderly, yet we were able to study mortality across all age groups. The fact that mortality is *not* raised in patients with a normal inflammatory marker suggests that the test result, rather than the clinician's decision to test, is significant.[12]

The main weakness is the lack of information about the reasons for testing; we cannot determine which tests were done for diagnosis, monitoring or non-specific purposes. However, this is also a strength, as it increases the generalisability of the results, which are not limited to specific subgroups of tested patients.

Comparison to previous literature

Several previous frailty indices have previously been developed, the most commonly used being the electronic frailty index (eFI)[13] and Qmortality.[14] The former has a c-statistic of 0.76; the latter a c-statistic of 0.85 for women and 0.84 for men. More recent research by Deelen *et al* has used combinations of biomarkers to predict mortality across all age groups; 226 potential biomarkers were selected, but CRP, ESR and PV were not considered.[15] They generated a model using 14 biomarkers with a c-statistic of 0.837: however, of the biomarkers considered, only albumin is

For peer review only

available in primary care, limiting the clinical usefulness of their findings. CRP by comparison is a low cost and widely available test. With a c-statistic of 0.78 for CRP alone, and 0.89 for a model including CRP, age and gender, inflammatory markers could be a simple indicator with a comparable accuracy to currently used mortality prediction tools.

The association between CRP and mortality is in keeping with population-based studies examining all-cause mortality[2 16] and cardiovascular mortality,[5] as well as hospital-based studies of patients with specific diseases including COPD,[17] diabetes,[18] chronic kidney disease,[8 19] pneumonia[6] and cancer.[20 21] The research reported here demonstrates that this association is also seen in a primary care setting, and over the shorter term. The finding that men with raised inflammatory markers are at higher mortality risk than women may reflect gender differences in healthcare-seeking behaviour in primary care; men have lower rates of consultation, so might be 'sicker' on average when receiving blood tests.

Previous research has shown limited diagnostic utility of inflammatory markers in a primary care setting, where sensitivity is low, false positives are common, and abnormal tests can lead to increased rates of GP consultations, tests and referrals.[9] The fact that inflammatory markers have a higher c-statistic for mortality than for cancer, infections or autoimmune diseases,[22] may reflect the fact that inflammatory markers have both diagnostic and prognostic utility for a broad range of pathologies.

Clinical implications

General practitioners should interpret raised inflammatory markers within the wider clinical context; where the cause of inflammation is identifiable and treatable, mortality risks should not cause undue alarm. The findings back up the current clinical practice of repeating an abnormal test; a subsequent normal result is reassuring with mortality risk reducing to near normal. However,

For peer review only

clinicians should consider whether older patients with a persistently raised inflammatory markers are reaching the end of life.

There is debate over the utility of predicting mortality,[23] given the lack of evidence-based interventions. It seems unlikely therefore, that clinicians would choose to test inflammatory markers purely for the purpose of mortality prediction, particularly given that false positives have been shown to lead to cascades of follow on tests, appointments and referrals.[9] However, where inflammatory marker test results are available, they may add useful information to improve prediction of mortality and assessment of frailty in primary care.

Unanswered questions and future research

Future research should consider the added value of CRP testing in combination with other risk factors, to improve prediction of mortality in primary care. Predicting mortality in itself, however, is not enough, as tests will only benefit patients if they influence management. Evidence based interventions for frailty must therefore be developed alongside predictive tools.

For peer review only

Footnotes

Patient consent for publication:

Not required

Contributors:

All authors contributed to conception and planning the study. JW analysed the data, and wrote the first draft of the paper. All authors contributed to subsequent drafts and read and approved the final manuscript.

Funding:

This report is independent research arising from JW's Doctoral Research Fellowship (DRF-2016-09-034) supported by the National Institute for Health Research.

JB, PW, CS and JW were supported by the National Institute for Health Research Applied Research Collaboration West (NIHR ARC West). The views expressed in this publication are those of the authors and not necessarily those of the NHS, the National Institute for Health Research, Health Education England or the Department of Health and Social Care.

Competing interests:

None declared

Data sharing statement:

This study is based on data from the Clinical Practice Research Datalink (CPRD) obtained under licence from the UK Medicines and Healthcare products Regulatory Agency, which does not permit data shaing. The data is provided by patients and collected by the NHS as part of their care and support. The interpretation and conclusions contained in this study are those of the authors alone.

Ethical approval

This study was approved by the Independent Scientific Application Committee for MHRA database research (ISAC) protocol number 17_003. The protocol is available at: https://www.cprd.com/protocol/diagnostic-utility-inflammatory-markers-primary-care-prospective-cohort-study

Patient and public involvement

Patients were not involved in the design, conduct, reporting or dissemination of this research

For peer review only

I, the Submitting Author has the right to grant and does grant on behalf of all authors of the Work (as defined in the below author licence), an exclusive licence and/or a non-exclusive licence for contributions from authors who are: i) UK Crown employees; ii) where BMJ has agreed a CC-BY licence shall apply, and/or iii) in accordance with the terms applicable for US Federal Government officers or employees acting as part of their official duties; on a worldwide, perpetual, irrevocable, royalty-free basis to BMJ Publishing Group Ltd ("BMJ") its licensees and where the relevant Journal is co-owned by BMJ to the co-owners of the Journal, to publish the Work in BMJ Open and any other BMJ products and to exploit all rights, as set out in our licence.

The Submitting Author accepts and understands that any supply made under these terms is made by BMJ to the Submitting Author unless you are acting as an employee on behalf of your employer or a postgraduate student of an affiliated institution which is paying any applicable article publishing charge ("APC") for Open Access articles. Where the Submitting Author wishes to make the Work available on an Open Access basis (and intends to pay the relevant APC), the terms of reuse of such Open Access shall be governed by a Creative Commons licence – details of these licences and which Creative Commons licence will apply to this Work are set out in our licence referred to above.

For peer review only

REFERENCES

1. Multimorbidity: clinical assessment and management (NICE clinical guideline 56): National Insitute for Health and Care Excellence. 2016.
2. Zacho J, Tybjaerg-Hansen A, Nordestgaard BG. C-reactive protein and all-cause mortality--the Copenhagen City Heart Study. *Eur Heart J* 2010;31(13):1624-32. doi: 10.1093/eurheartj/ehq103 [published Online First: 2010/04/29]
3. Strandberg TE, Tilvis RS. C-reactive protein, cardiovascular risk factors, and mortality in a prospective study in the elderly. *Arteriosclerosis, thrombosis, and vascular biology* 2000;20(4):1057-60. [published Online First: 2000/04/15]
4. Barron E, Lara J, White M, et al. Blood-borne biomarkers of mortality risk: systematic review of cohort studies. *PloS one* 2015;10(6):e0127550. doi: 10.1371/journal.pone.0127550 [published Online First: 2015/06/04]
5. Kaptoge S, Di Angelantonio E, Lowe G, et al. C-reactive protein concentration and risk of coronary heart disease, stroke, and mortality: an individual participant meta-analysis. *Lancet* 2010;375(9709):132-40. doi: 10.1016/s0140-6736(09)61717-7
6. Viasus D, Del Rio-Pertuz G, Simonetti AF, et al. Biomarkers for predicting short-term mortality in community-acquired pneumonia: A systematic review and meta-analysis. *The Journal of infection* 2016;72(3):273-82. doi: 10.1016/j.jinf.2016.01.002 [published Online First: 2016/01/19]
7. Gradel KO, Thomsen RW, Lundbye-Christensen S, et al. Baseline C-reactive protein level as a predictor of mortality in bacteraemia patients: a population-based cohort study. *Clinical Microbiology and Infection* 2011;17(4):627-32. doi: https://doi.org/10.1111/j.1469-0691.2010.03284.x
8. Li WJ, Chen XM, Nie XY, et al. Cardiac troponin and C-reactive protein for predicting all-cause and cardiovascular mortality in patients with chronic kidney disease: a meta-analysis. *Clinics (Sao Paulo, Brazil)* 2015;70(4):301-11. doi: 10.6061/clinics/2015(04)14 [published Online First: 2015/05/29]
9. Watson J SC, Whiting P, Banks J, Pyne Y, Hamilton W. Added value and cascade effects of inflammatory marker tests in UK primary care: a cohort study from the Clinical Practice Research Datalink. *BJGP* 2019; 69 (684): e470-e478. DOI: https://doi.org/10.3399/bjgp19X704321
10. DeLong ER, DeLong DM, Clarke-Pearson DLJB. Comparing the areas under two or more correlated receiver operating characteristic curves: a nonparametric approach. 1988:837-45.
11. Stata Statistical Software: Release 15. [program]: College Station, TX: StataCorp LLC, 2017.
12. Watson J BS, Hamilton F, Hamilton W, Mounce L. Lessons from biases in electronic health record data: the importance of clinical vigilance with negative test results. *BMJ* 2018;361. Available at: https://www.bmj.com/content/361/bmj.k1479/rr-0
13. Clegg A, Bates C, Young J, et al. Development and validation of an electronic frailty index using routine primary care electronic health record data. *Age and Ageing* 2016;45(3):353-60. doi: 10.1093/ageing/afw039 %J Age and Ageing
14. Hippisley-Cox J, Coupland C. Development and validation of QMortality risk prediction algorithm to estimate short term risk of death and assess frailty: cohort study. 2017;358:j4208. doi: 10.1136/bmj.j4208 %J BMJ
15. Deelen J, Kettunen J, Fischer K, et al. A metabolic profile of all-cause mortality risk identified in an observational study of 44,168 individuals. *Nature Communications* 2019;10(1):3346. doi: 10.1038/s41467-019-11311-9
16. Li Y, Zhong X, Cheng G, et al. Hs-CRP and all-cause, cardiovascular, and cancer mortality risk: a meta-analysis. *Atherosclerosis* 2017;259:75-82.

For peer review only

17. Ford ES, Cunningham TJ, Mannino DM. Inflammatory markers and mortality among US adults with obstructive lung function. *Respirology* 2015;20(4):587-93. doi: 10.1111/resp.12499 [published Online First: 2015/03/04]
18. Landman GW, Kleefstra N, Groenier KH, et al. Inflammation biomarkers and mortality prediction in patients with type 2 diabetes (ZODIAC-27). *Atherosclerosis* 2016;250:46-51. doi: 10.1016/j.atherosclerosis.2016.04.015 [published Online First: 2016/05/15]
19. Zhang W, He J, Zhang F, et al. Prognostic role of C-reactive protein and interleukin-6 in dialysis patients: a systematic review and meta-analysis. *Journal of nephrology* 2013;26(2):243-53. doi: 10.5301/jn.5000169 [published Online First: 2012/06/12]
20. Jing X, Huang C, Zhou H, et al. Association between serum C-reactive protein value and prognosis of patients with non-small cell lung cancer: a meta-analysis. *International journal of clinical and experimental medicine* 2015;8(7):10633-9. [published Online First: 2015/09/18]
21. Shrotriya S, Walsh D, Bennani-Baiti N, et al. C-Reactive Protein Is an Important Biomarker for Prognosis Tumor Recurrence and Treatment Response in Adult Solid Tumors: A Systematic Review. *PloS one* 2015;10(12):e0143080. doi: 10.1371/journal.pone.0143080 [published Online First: 2015/12/31]
22. Watson J JH, Salisbury C, Whiting P, Banks J, Hamilton W. Use of multiple inflammatory marker tests in primary care: using Clinical Practice Research Datalink to evaluate accuracy. *BJGP* 2019; 69 (684): e462-e469. DOI: https://doi.org/10.3399/bjgp19X704309
23. Hamilton W, Round J. Identifying frailty in primary care. 2017;358:j4478. doi: 10.1136/bmj.j4478 %J BMJ

For peer review only

Figure 1: Flowchart of one-year mortality (95% confidence intervals) according to CRP test results. The right-hand column shows one-year mortality according to repeat test result; defined as the first CRP test performed in the 3 months following the index date

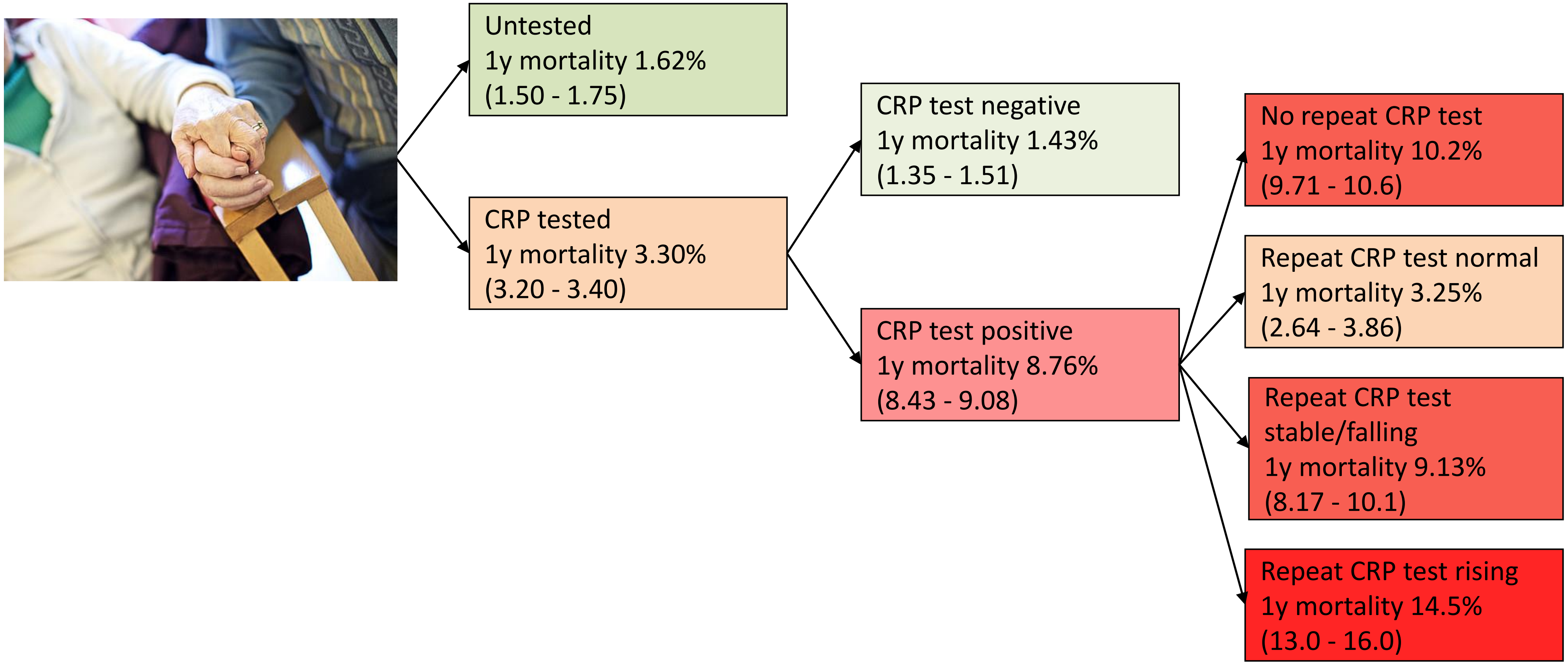

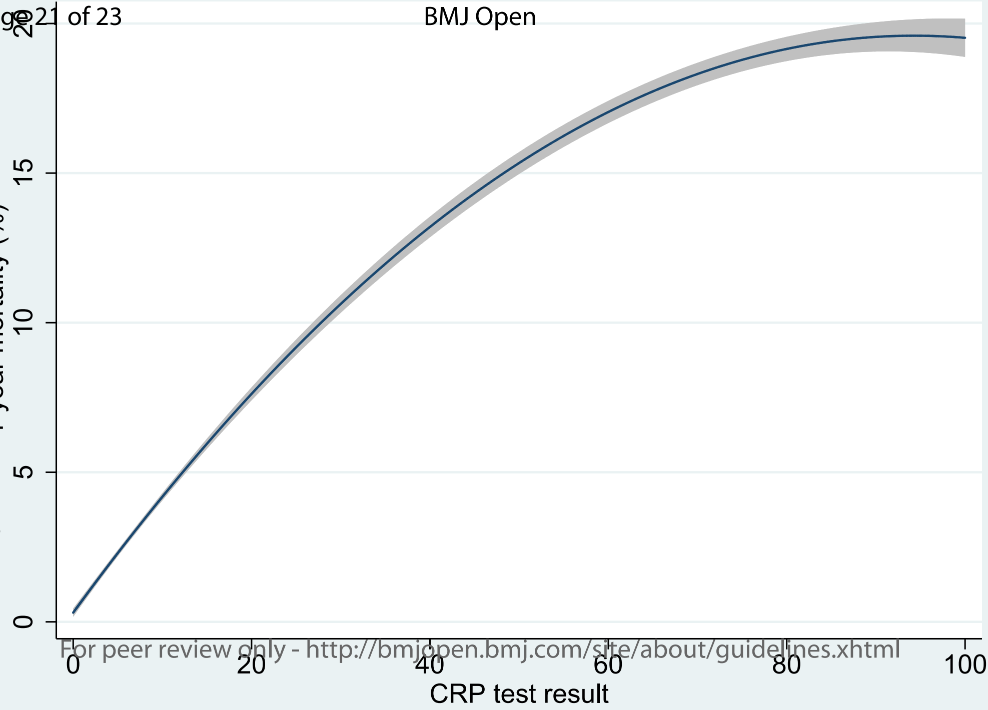

For peer review only - http://bmjopen.bmj.com/site/about/guidelines.xhtml

Section & Topic	No	Item	Reported on page #
**TITLE OR ABSTRACT**			
	1	Identification as a study of diagnostic accuracy using at least one measure of accuracy (such as sensitivity, specificity, predictive values, or AUC)	Abstract page 2-3
**ABSTRACT**			
	2	Structured summary of study design, methods, results, and conclusions (for specific guidance, see STARD for Abstracts)	Abstract Page 2-3
**INTRODUCTION**			
	3	Scientific and clinical background, including the intended use and clinical role of the index test	5
	4	Study objectives and hypotheses	5
**METHODS**			
Study design	5	Whether data collection was planned before the index test and reference standard were performed (prospective study) or after (retrospective study)	6
Participants	6	Eligibility criteria	5-6
	7	On what basis potentially eligible participants were identified (such as symptoms, results from previous tests, inclusion in registry)	5-6
	8	Where and when potentially eligible participants were identified (setting, location and dates)	5-6
	9	Whether participants formed a consecutive, random or convenience series	5-6
Test methods	10a	Index test, in sufficient detail to allow replication	6
	10b	Reference standard, in sufficient detail to allow replication	6
	11	Rationale for choosing the reference standard (if alternatives exist)	n/a
	12a	Definition of and rationale for test positivity cut-offs or result categories of the index test, distinguishing pre-specified from exploratory	6
	12b	Definition of and rationale for test positivity cut-offs or result categories of the reference standard, distinguishing pre-specified from exploratory	n/a
	13a	Whether clinical information and reference standard results were available to the performers/readers of the index test	n/a
	13b	Whether clinical information and index test results were available to the assessors of the reference standard	n/a
Analysis	14	Methods for estimating or comparing measures of diagnostic accuracy	6
	15	How indeterminate index test or reference standard results were handled	n/a
	16	How missing data on the index test and reference standard were handled	6
	17	Any analyses of variability in diagnostic accuracy, distinguishing pre-specified from exploratory	
	18	Intended sample size and how it was determined	Sample size calculations are provided in linked paper which is referenced
**RESULTS**			
Participants	19	Flow of participants, using a diagram	In linked paper
	20	Baseline demographic and clinical characteristics of participants	Table 1 page 7
	21a	Distribution of severity of disease in those with the target condition	n/a (target condition = death)
	21b	Distribution of alternative diagnoses in those without the target condition	n/a
	22	Time interval and any clinical interventions between index test and reference standard	1 year mortality
Test results	23	Cross tabulation of the index test results (or their distribution) by the results of the reference standard	
	24	Estimates of diagnostic accuracy and their precision (such as 95% confidence intervals)	Table 2
	25	Any adverse events from performing the index test or the reference standard	n/a - observational
**DISCUSSION**			
	26	Study limitations, including sources of potential bias, statistical uncertainty, and generalisability	9
	27	Implications for practice, including the intended use and clinical role of the index test	10 - 11

For peer review only - http://bmjopen.bmj.com/site/about/guidelines.xhtml

**OTHER INFORMATION**			
	**28**	Registration number and name of registry	15
	**29**	Where the full study protocol can be accessed	15
	**30**	Sources of funding and other support; role of funders	15

For peer review only

STARD 2015

AIM

STARD stands for "Standards for Reporting Diagnostic accuracy studies". This list of items was developed to contribute to the completeness and transparency of reporting of diagnostic accuracy studies. Authors can use the list to write informative study reports. Editors and peer-reviewers can use it to evaluate whether the information has been included in manuscripts submitted for publication.

EXPLANATION

A **diagnostic accuracy study** evaluates the ability of one or more medical tests to correctly classify study participants as having a **target condition.** This can be a disease, a disease stage, response or benefit from therapy, or an event or condition in the future. A medical test can be an imaging procedure, a laboratory test, elements from history and physical examination, a combination of these, or any other method for collecting information about the current health status of a patient.

The test whose accuracy is evaluated is called **index test.** A study can evaluate the accuracy of one or more index tests. Evaluating the ability of a medical test to correctly classify patients is typically done by comparing the distribution of the index test results with those of the **reference standard**. The reference standard is the best available method for establishing the presence or absence of the target condition. An accuracy study can rely on one or more reference standards.

If test results are categorized as either positive or negative, the cross tabulation of the index test results against those of the reference standard can be used to estimate the **sensitivity** of the index test (the proportion of participants *with* the target condition who have a positive index test), and its **specificity** (the proportion *without* the target condition who have a negative index test). From this cross tabulation (sometimes referred to as the contingency or "2x2" table), several other accuracy statistics can be estimated, such as the positive and negative **predictive values** of the test. Confidence intervals around estimates of accuracy can then be calculated to quantify the statistical **precision** of the measurements.

If the index test results can take more than two values, categorization of test results as positive or negative requires a **test positivity cut-off**. When multiple such cut-offs can be defined, authors can report a receiver operating characteristic (ROC) curve which graphically represents the combination of sensitivity and specificity for each possible test positivity cut-off. The **area under the ROC curve** informs in a single numerical value about the overall diagnostic accuracy of the index test.

The **intended use** of a medical test can be diagnosis, screening, staging, monitoring, surveillance, prediction or prognosis. The **clinical role** of a test explains its position relative to existing tests in the clinical pathway. A replacement test, for example, replaces an existing test. A triage test is used before an existing test; an add-on test is used after an existing test.

Besides diagnostic accuracy, several other outcomes and statistics may be relevant in the evaluation of medical tests. Medical tests can also be used to classify patients for purposes other than diagnosis, such as staging or prognosis. The STARD list was not explicitly developed for these other outcomes, statistics, and study types, although most STARD items would still apply.

DEVELOPMENT

This STARD list was released in 2015. The 30 items were identified by an international expert group of methodologists, researchers, and editors. The guiding principle in the development of STARD was to select items that, when reported, would help readers to judge the potential for bias in the study, to appraise the applicability of the study findings and the validity of conclusions and recommendations. The list represents an update of the first version, which was published in 2003.

More information can be found on [http://www.equator-network.org/reporting-guidelines/stard.](http://www.equator-network.org/reporting-guidelines/stard)

CC BY SA

BMJ Open

Raised inflammatory markers as a predictor of one-year mortality: A cohort study using primary care electronic health record data

Journal:	*BMJ Open*
Manuscript ID	bmjopen-2019-036027.R1
Article Type:	Original research
Date Submitted by the Author:	26-Mar-2020

Complete List of Authors:	Watson, Jessica; University of Bristol, Centre for Academic Primary Care<br>Whiting, Penny; University of Bristol, School of Social and Community Medicine<br>Salisbury, Chris; University of Bristol, Academic Unit of Primary Health Care<br>Banks, Jonathan; University of Bristol, Social & Community Medicine<br>Hamilton, Willie; University of Exeter Medical School, Primary Care Diagnostics

**Primary Subject Heading**:	General practice / Family practice
Secondary Subject Heading:	Epidemiology, Geriatric medicine
Keywords:	PRIMARY CARE, EPIDEMIOLOGY, Chemical pathology < PATHOLOGY, GERIATRIC MEDICINE

SCHOLARONE™
Manuscripts

I, the Submitting Author has the right to grant and does grant on behalf of all authors of the Work (as defined in the below author licence), an exclusive licence and/or a non-exclusive licence for contributions from authors who are: i) UK Crown employees; ii) where BMJ has agreed a CC-BY licence shall apply, and/or iii) in accordance with the terms applicable for US Federal Government officers or employees acting as part of their official duties; on a worldwide, perpetual, irrevocable, royalty-free basis to BMJ Publishing Group Ltd ("BMJ") its licensees and where the relevant Journal is co-owned by BMJ to the co-owners of the Journal, to publish the Work in this journal and any other BMJ products and to exploit all rights, as set out in our licence.

The Submitting Author accepts and understands that any supply made under these terms is made by BMJ to the Submitting Author unless you are acting as an employee on behalf of your employer or a postgraduate student of an affiliated institution which is paying any applicable article publishing charge ("APC") for Open Access articles. Where the Submitting Author wishes to make the Work available on an Open Access basis (and intends to pay the relevant APC), the terms of reuse of such Open Access shall be governed by a Creative Commons licence – details of these licences and which Creative Commons licence will apply to this Work are set out in our licence referred to above.

Other than as permitted in any relevant BMJ Author's Self Archiving Policies, I confirm this Work has not been accepted for publication elsewhere, is not being considered for publication elsewhere and does not duplicate material already published. I confirm all authors consent to publication of this Work and authorise the granting of this licence.

For peer review only

Raised inflammatory markers as a predictor of one-year mortality: A cohort study using primary care electronic health record data

Jessica Watson[1,2], Penny Whiting[1,2], Chris Salisbury[1,2], Jonathan Banks[1,2], Willie Hamilton[3]

1. Centre for Academic Primary Care, Department of Population Health Sciences, Bristol Medical School, University of Bristol, Bristol, UK
2. NIHR CLAHRC West, Bristol, UK
3. University of Exeter Medical School, Exeter, UK

Corresponding author:

Dr Jessica Watson

Centre for Academic Primary Care, Department of Population Health Sciences

Canynge Hall

39 Whatley Road

Bristol, BS8 2PS

Jessica.Watson@bristol.ac.uk

Tel: (+44) 07837002664

Word count: 2,094 (excluding abstract, references, figures and tables)

For peer review only

Abstract

Objectives

Identification of patients at increased mortality risk is important in the context of increasing multimorbidity and an ageing population, to help facilitate the planning and delivery of services. The aim of this study was to examine one-year all-cause mortality in a cohort of primary care patients in whom inflammatory markers including C-reactive protein (CRP), erythrocyte sedimentation rate (ESR) and plasma viscosity (PV), had been tested.

Design

Observational cohort study using GP Electronic Health Records from the Clinical Practice Research Datalink, with linkage to ONS Death Registry.

Setting

UK Primary Care

Participants

159,325 patients with inflammatory marker tests done in 2014 and 39,928 age, sex and practice matched controls without inflammatory marker testing. ONS Death registry data was available for 109,966 participants.

Primary and secondary outcome measures

One-year mortality in those with raised inflammatory markers compared to normal inflammatory markers and untested controls. Sub-analyses stratified one-year mortality by age-group, gender and cause of death.

For peer review only

Results

Patients with a raised inflammatory marker (n=47,797) had an overall one-year all-cause mortality of 6.89%, compared to 1.41% in those with normal inflammatory markers (p<0.001) and 1.62% in untested controls. A raised CRP is associated with the highest mortality rate at 8.76% compared to 4.99% for ESR and 4.66% for PV. One-year mortality is higher in men with a raised inflammatory marker compared to women (9.78% versus 5.29%). The c-statistic of a simple mortality prediction model containing age, sex and CRP test result is 0.89.

Conclusions

Inflammatory markers are a strong predictor of all-cause mortality in primary care, with a c-statistic comparable to several previously developed frailty indices. Future research should consider the added value of CRP testing, in combination with other risk factors, to improve prediction of mortality in primary care. Evidence based interventions for frailty are needed alongside predictive tools.

For peer review only

Strengths and limitations of this study

- Using a large observational primary care electronic health records dataset we demonstrate a strong association between raised inflammatory markers and one-year mortality
- As test results were transferred electronically to the GP record, there is a very low risk of transcription error or bias.
- Use of ONS death registry data increased the accuracy of recording of mortality, the primary outcome.
- Previous studies have focussed on predictors of mortality in the elderly, yet we were able to study mortality across all age groups.
- Future research should consider the added value of CRP testing in combination with other risk factors, to improve prediction of mortality in primary care

For peer review only

Background

Identification of patients at increased risk of mortality is important in the context of increasing multimorbidity and an ageing population, with the aim of helping facilitate the planning and delivery of services. Multiple risk tools have therefore been developed to predict mortality; these can be used to help predict frailty, unplanned hospital admissions and to allow targeted interventions to people at an increased mortality risk. The National Institute for Health and Care Excellence (NICE) multimorbidity guidelines systematically reviewed 41 of these mortality risk tools; the majority were of low or very low quality and a need for further research in this area was identified.[1] Current risk tools include variables such as disease status, sociodemographic factors and laboratory test results (eg anaemia, raised platelets), however, none in current use include an inflammatory marker test.

Inflammatory markers such as C reactive protein (CRP), erythrocyte sedimentation rate (ESR) and plasma viscosity (PV) are commonly used in primary care to aid diagnosis and monitoring of infections and inflammatory conditions. Cohort studies in the general population have reported inflammatory markers as predicting future mortality,[2-4] particularly from cardiovascular disease.[5] CRP has also been shown to have predictive value for mortality in hospital settings.[6-8] The clinical relevance of these findings in primary care settings, and over the shorter term, has not previously been described.

The aim of this study was to examine one-year all-cause mortality in a cohort of primary care patients in whom inflammatory marker bloods had been tested.

Methods

This was a secondary analysis of an observational cohort study of 160,000 patients aged >18 from the Clinical Practice Research Datalink (CPRD) with inflammatory marker blood testing in 2014, and

For peer review only

40,000 age, sex and practice matched controls without inflammatory marker testing also in 2014. The methods have been described fully in previous paired papers describing the disease outcomes following inflammatory marker testing.[9 10] Patients were excluded from the analysis if the inflammatory marker test result was missing (n=673) or if results were so abnormal as to be considered spurious (n=2). Linkage to ONS death registry data was available for 109,966. The three inflammatory marker tests studied were C-reactive protein (CRP), erythrocyte sedimentation rate (ESR) and plasma viscosity (PV). We defined a raised inflammatory marker using the mean upper limit of normal for laboratories within our study. For CRP this was 6.8mg/l, for simplicity rounded to 7mg/l; for PV 1.72mPa.s. For ESR this mean upper limit of normal was rounded and stratified by gender and age.[9] When the same inflammatory marker was repeated on the same day (n=231), we retained the highest value. The index date was defined as the first date of inflammatory marker testing in 2014, with one-year mortality defined as death within one year of this index date. Date of death was defined as the earlier date of recorded death in either CPRD or ONS death registry. Cause of death was available from death certification data in 3,141 out of 5,512 deaths where ONS linkage was available.

Analysis

The primary analysis compared one-year mortality in those with raised versus normal inflammatory markers and compared to untested controls. Sub-analyses stratified one-year mortality by age group and gender and cause of death. For each of the three tests (CRP, ESR and PV), dichotomised test results were cross-classified with the reference standard one-year mortality, allowing sensitivity and specificity to be calculated. Logistic regression was used to calculate diagnostic odds ratios, with and without adjustment for age and gender.

Test results were also analysed on a continuous scale, using logistic regression to determine the dose response relationship between inflammatory marker test result and mortality. The area under receiver operator curve (AUC), otherwise known as the c-statistic, was calculated using a logistic

For peer review only

regression model using test result on a continuous scale, with log transformation due to the skewed nature of the data, with and without age and gender as covariates. Comparisons of the c-statistic were made using DeLong method.[11] All analyses were done using Stata15.[12]

Patients were not involved in the design, conduct, reporting or dissemination of this research.

Results

Demographics of the tested cohort and untested controls, compared to the UK population are shown in **supplementary table 1**. After exclusions there were 159,325 tested patients of whom 114,198 (71.7%) had a CRP test, 92,325 (58.0%) an ESR test, and 15,994 (10%) a PV test; 62,789 (39.4%) had more than one inflammatory marker performed simultaneously, mostly CRP and ESR together (51,546). Overall 47,797 (30%) had one or more raised inflammatory marker on the index date. In total 5,512 patients died within one-year of the index date; 648 deaths in the untested group, 1,572 deaths in the normal inflammatory marker group and 3,292 deaths in the group with one or more raised inflammatory marker.

Overall mortality rates (Table 1)

Patients with a raised inflammatory marker (n=47,797) had an overall one-year mortality of 6.89%, compared to 1.41% in those with normal inflammatory markers ($p<0.001$). In the untested comparison cohort, one-year mortality was 1.62%. The association between raised inflammatory markers and one-year mortality was seen in all age groups apart from the under 30-year olds. In older age groups the absolute increase in risk was considerable; a raised inflammatory marker in the over 80s was associated with a one-year mortality of 21.8%, compared to 8.6% in the over 80s with normal inflammatory markers.

For peer review only

Men with a raised inflammatory marker had a significantly higher one-year mortality rate than women with a raised inflammatory marker (9.78% versus 5.29%). Patients with a raised CRP had a one-year mortality of 8.76% compared to 4.99% for those with raised ESR and 4.66% for raised PV.

In the 62,789 patients with more than one inflammatory marker performed simultaneously on the index date, one-year mortality was higher in the 9,029 patients with concordant raised values at 6.9%, compared to the 13,783 with discordant results (one raised, one normal) who had a one-year mortality of 2.8%. In the 39,977 patients with two simultaneous negative inflammatory markers one-year mortality was 0.85%.

For peer review only

Table 1: One-year mortality (%, 95% CI) subdivided by age, gender and test result

	Untested (n=39,928)	Normal inflammatory markers (n=111,528)	Any raised inflammatory marker (n=47,797)	Raised CRP (n=29,164)	Raised ESR (n=23,138)	Raised PV (n=4,568)
Overall (n=199,253)	1.62 (1.50-1.75)	1.41 (1.34-1.48)	6.89 (6.66-7.11)	8.76 (8.43-9.08)	4.99 (4.71-5.27)	4.66 (4.05-5.27)
Age						
<30 (n=21,732)	0.09 (0.02-0.18)	0.04 (0.01-0.08)	0.08 (0.00-0.17)	0.13 (0.00-0.27)	0.00 (0.00-0.00)	0.0 (0.00-0.00)
30-39 (n=22,718)	0.04 (0.00-0.10)	0.08 (0.03-0.13)	0.37 (0.19-5.47)	0.50 (0.23-0.77)	0.28 (0.06-0.51)	0.0 (0.00-0.00)
40-49 (n=31,588)	0.19 (0.08-0.30)	0.15 (0.09-0.20)	1.12 (0.86-1.39)	1.54 (1.14-1.95)	0.92 (0.59-1.25)	0.90 (0.01-1.69)
50-59 (n=35,044)	0.41 (0.26-0.56)	0.41 (0.32-0.49)	2.37 (2.03-2.70)	2.98 (2.48-3.47)	1.79 (1.38-2.21)	1.62 (0.75-2.50)
60-69 (n=35,094)	0.84 (0.62-1.05)	0.91 (0.78-1.05)	4.96 (4.51-5.40)	6.72 (6.05-7.39)	3.88 (3.33-4.43)	3.27 (2.12-4.42)
70-79 (30,251)	2.27 (1.90-2.64)	2.51 (2.26-2.76)	9.39 (8.77-10.0)	11.38 (10.5-12.2)	7.35 (6.54-8.16)	6.32 (4.75-7.88)
>80 (22,826)	8.88 (8.05-9.70)	8.61 (8.07-9.16)	21.8 (20.9-22.7)	25.9 (24.7-27.1)	16.1 (14.9-17.3)	15.6 (12.9-18.3)
Gender						
Male (n=75,787)	1.86 (1.64-2.07)	1.58 (1.46-1.70)	9.78 (9.33-10.2)	11.48 (10.9-12.1)	7.98 (7.35-8.61)	6.63 (5.44-7.81)
Female (n=123,466)	1.48 (1.33-1.63)	1.30 (1.22-1.39)	5.29 (5.04-5.54)	6.99 (6.61-7.36)	3.65 3.36-3.94)	3.51 (2.84-4.18)

Table 2 shows the performance characteristics of inflammatory markers, including sensitivity, specificity and c-statistic. CRP had the highest sensitivity of the three tests at 67.8% and the greatest c-statistic at 0.78. Odds ratios (OR) reduced after adjustment for age and gender but were still significant with an adjusted OR for a raised CRP of 4.5 ($p<0.001$), 2.9 for raised ESR and 2.1 for raised PV.

A logistic regression model containing age (as a continuous variable) and gender had a c-statistic of 0.85, compared to 0.89 for a full model containing age, gender and CRP test result as a continuous variable ($p < 0.001$); 0.88 with age, gender and ESR ($p < 0.001$); and 0.87 with age gender and PV ($p < 0.001$).

For peer review only

Table 2: Performance characteristics of CRP, ESR and PV for predicting one-year mortality

				Univariable logistic regression		Adjusted for age and gender	
	Sensitivity	Specificity	C-statistic*	OR	P	OR	P
**CRP**	67.8% (66.3-69.3)	75.9% (75.6-76.2)	0.78 (0.77-0.78)	6.6 (6.2-7.1)	<0.001	4.5 (4.2-4.8)	<0.001
**ESR**	56.6% (54.4-58.7)	75.7% (75.4-75.9)	0.66 (0.65-0.67	4.1 (3.7-4.4)	<0.001	2.9 (2.7-3.2)	<0.001
**PV**	52.0% (47.0-56.9)	72.1% (71.3-72.8)	0.62 (0.60-0.64)	2.8 (2.3-3.4)	<0.001	2.1 (1.7-2.6)	<0.001

*C-statistic calculated using log transformed test results as a continuous variable

For peer review only

Repeat testing

Figure 1 shows the one-year mortality in patients according to the subsequent repeat inflammatory marker results, using the most common test performed; CRP. The fact that a CRP test was requested by a GP was in itself, predictive of increased mortality, with one-year mortality of 3.3% in the tested versus 1.6% in the untested cohort. This increased to 8.76% one-year mortality if a single CRP test was raised, 9.13% if a second test was persistently raised and 14.5% if the second test was raised further still. Those with a raised inflammatory marker which was not subsequently rechecked had a one-year mortality rate of 10.2%, compared to 3.25% if a subsequent CRP normalised.

Figure 1: Flowchart of one-year mortality (95% confidence intervals) according to CRP test results. The right-hand column shows one-year mortality according to repeat test result; defined as the first CRP test performed in the 3 months following the index date

Dose response relationship

A dose response relationship was found between result of the index CRP test as a continuous variable and one-year mortality (figure 2). In 2,184 people with a CRP >=100mg/L overall one-year

mortality was 20.2%. Similar associations, with wider confidence intervals, were found for ESR and PV (not shown).

Figure 2: Polynomial logistic regression of mortality against CRP test result as a continuous variable

Cause of death

Cause of death from ONS death certification was available for 3,141 out of 5,512 total deaths in the cohort. **Table 3** summarises the cause of death amongst patients with raised inflammatory markers, compared to those with normal inflammatory markers, and untested controls. The commonest cause of death in the 26,507 patients with raised inflammatory markers was cancer (696 deaths) followed by cardiovascular disease (449 deaths). Odds of mortality in the raised versus normal inflammatory marker groups was highest for cancer (adjusted OR 6.34), followed by infections (adjusted OR 4.11). However significant increased odds of mortality were seen for all disease categories with the exception of deaths due to falls, musculoskeletal causes and senility. **Supplementary table 2** shows cause of death by age group for patients with raised inflammatory markers; cancer was the commonest cause of death in 40-79 year olds, cardiovascular disease increased with age and was the commonest cause of death in the over 80 age group.

For peer review only

Table 3: Cause of death amongst patients with ONS death registry linkage (n=109,966)

Cause of death	Untested controls (n=22,069)		Normal inflammatory markers (n=61,390)		Raised inflammatory markers (n=26,507)		Comparison between normal and raised inflammatory markers	
	Number of deaths	1 year mortality (%)	Number of deaths	1 year mortality (%)	Number of deaths	1 year mortality (%)	Unadjusted odds ratio (CI)	Odds ratio, adjusted for age and gender (CI)
All-cause mortality	380	1.74	889	1.45	1,872	7.08	5.16* (4.76-5.60)	3.66* (3.37-3.99)
Cancer	86	0.39	195	0.32	696	2.63	8.46* (7.21-9.93)	6.34* (5.40-7.46)
Cardiovascular disease	115	0.52	295	0.48	449	1.69	3.57* (3.08-4.14)	2.18* (1.87- 2.54)
Respiratory	53	0.24	141	0.23	264	1.00	4.37* (3.56-5.36)	2.68* (2.18-3.30)
Dementia	38	0.17	69	0.11	119	0.45	4.01* (2.98-5.39)	2.21* (1.64-2.99)
Gastrointestinal	16	0.07	42	0.07	86	0.32	4.75* (3.29-6.88)	3.58* (2.46-5.20)
Genitourinary	8	0.04	16	0.03	39	0.15	5.65* (3.16-10.1)	3.13* (1.74-5.64)
Infection	5	0.02	11	0.02	29	0.11	6.11* (3.05-12.2)	4.08* (2.03-8.23)
Blood disorder	2	0.01	11	0.02	25	0.09	5.27* (2.59-10.7)	3.11 (1.52-6.37)
Senility	12	0.05	8	0.01	21	0.08	6.08* (2.69-13.7)	2.80 (1.23-6.38)
Musculoskeletal	3	0.01	8	0.01	14	0.05	4.05* (1.70-9.67)	2.40 (1.00-5.77)
Falls	4	0.02	10	0.02	6	0.02	1.39 (0.51-3.82)	0.80 (0.29-2.22)
Other	38	0.17	83	0.14	124	0.47	3.47* (2.62-4.59)	2.41* (1.82-3.20)

*p<0.001

For peer review only

Discussion

Inflammatory markers are a strong predictor of all-cause mortality in primary care. The association between raised inflammatory markers and all-cause mortality is seen in all age groups except patients aged less than thirty years. Men with raised inflammatory markers have a higher one-year mortality than women (9.78% vs 5.29%). Of the three tests examined, CRP has the highest predictive accuracy for mortality. The overall c-statistic of a model containing age, sex and CRP test result of

0.89 is comparable to several previously developed frailty indices. Inflammatory markers could potentially be a simple indicator to improve prediction of life-expectancy in primary care.

Strengths and limitations

The major strength of this study is its size and its setting in primary care, making results relevant to clinical practice. As test results were transferred electronically to the GP record, there is a very low risk of transcription error or bias. Use of ONS death registry data increased the accuracy of recording of mortality, the primary outcome. Previous studies have focussed on predictors of mortality in the elderly, yet we were able to study mortality across all age groups. The fact that mortality is *not* raised in patients with a normal inflammatory marker suggests that the test result, rather than the clinician's decision to test, is significant.[13]

The main weakness is the lack of information about the reasons for testing; we cannot determine which tests were done for diagnosis, monitoring or non-specific purposes. However, this is also a strength, as it increases the generalisability of the results, which are not limited to specific subgroups of tested patients.

Comparison to previous literature

Several previous frailty indices have previously been developed, the most commonly used being the electronic frailty index (eFI)[14] and Qmortality.[15] The former has a c-statistic of 0.76; the latter a c-statistic of 0.85 for women and 0.84 for men. More recent research by Deelen *et al* has used combinations of biomarkers to predict mortality across all age groups; 226 potential biomarkers were selected, but CRP, ESR and PV were not considered.[16] They generated a model using 14 biomarkers with a c-statistic of 0.837: however, of the biomarkers considered, only albumin is

For peer review only

available in primary care, limiting the clinical usefulness of their findings. CRP by comparison is a low cost and widely available test. With a c-statistic of 0.78 for CRP alone, and 0.89 for a model including CRP, age and gender, inflammatory markers could be a simple indicator with a comparable accuracy to currently used mortality prediction tools.

The association between CRP and mortality is in keeping with population-based studies examining all-cause mortality[2 17] and cardiovascular mortality,[5] as well as hospital-based studies of patients with specific diseases including COPD,[18] diabetes,[19] chronic kidney disease,[8 20] pneumonia[6] and cancer.[21 22] The research reported here demonstrates that this association is also seen in a primary care setting, and over the shorter term. The finding that men with raised inflammatory markers are at higher mortality risk than women may reflect gender differences in healthcare-seeking behaviour in primary care; men have lower rates of consultation, so might be 'sicker' on average when presenting for blood tests.

Previous research has shown limited diagnostic utility of inflammatory markers in a primary care setting, where sensitivity is low, false positives are common, and abnormal tests can lead to increased rates of GP consultations, tests and referrals.[9] The fact that inflammatory markers have a higher c-statistic for mortality than for cancer, infections or autoimmune diseases,[10] may reflect the fact that inflammatory markers have both diagnostic and prognostic utility for a broad range of pathologies.

Clinical implications

General practitioners should interpret raised inflammatory markers within the wider clinical context; where the cause of inflammation is identifiable and treatable, mortality risks should not cause undue alarm. The findings back up the current clinical practice of repeating an abnormal test; a subsequent normal result is reassuring with mortality risk reducing to near normal. However,

For peer review only

clinicians should consider whether older patients with a persistently raised inflammatory markers are reaching the end of life.

There is debate over the utility of predicting mortality,[23] given the lack of evidence-based interventions. We would not recommend that clinicians test inflammatory markers purely for the purpose of mortality prediction, particularly given that false positives have been shown to lead to cascades of follow on tests, appointments and referrals.[9] However, general practitioners are already required to identify patients who are frail,[24] and inflammatory marker tests are commonly performed for many other reasons. Inflammatory marker test results, when available, may therefore add useful information to improve prediction of mortality and assessment of frailty in primary care.

Unanswered questions and future research

Future research should consider the added value of CRP testing in combination with other risk factors, to improve prediction of mortality in primary care. Predicting mortality in itself, however, is not enough, as tests will only benefit patients if they influence management. Evidence based interventions for frailty must therefore be developed alongside predictive tools.

For peer review only

Footnotes

Patient consent for publication:

Not required

Contributors:

WH, CS, PW, JB and JW all contributed to conception, design and planning of the study. JW analysed the data as part of her Doctoral Research Fellowship, with oversight of the conduct of the study provided by her PhD supervisors WH, CS, PW and JB. JW wrote the first draft of the paper. WH, CS, PW, JB and JW all contributed to subsequent drafts and read and approved the final manuscript.

Funding:

This report is independent research arising from JW's Doctoral Research Fellowship (DRF-2016-09-034) supported by the National Institute for Health Research.

JB, PW, CS and JW were supported by the National Institute for Health Research Applied Research Collaboration West (NIHR ARC West). CS is an NIHR Senior Investigator. The views expressed in this publication are those of the authors and not necessarily those of the NHS, the National Institute for Health Research, Health Education England or the Department of Health and Social Care.

Competing interests:

None declared

Data sharing statement:

This study is based on data from the Clinical Practice Research Datalink (CPRD) obtained under licence from the UK Medicines and Healthcare products Regulatory Agency, which does not permit data sharing. The data is provided by patients and collected by the NHS as part of their care and support. The interpretation and conclusions contained in this study are those of the authors alone.

Ethical approval

This study was approved by the Independent Scientific Application Committee for MHRA database research (ISAC) protocol number 17_003. The protocol is available at: https://www.cprd.com/protocol/diagnostic-utility-inflammatory-markers-primary-care-prospective-cohort-study

Patient and public involvement

Patients were not involved in the design, conduct, reporting or dissemination of this research

For peer review only

I, the Submitting Author has the right to grant and does grant on behalf of all authors of the Work (as defined in the below author licence), an exclusive licence and/or a non-exclusive licence for contributions from authors who are: i) UK Crown employees; ii) where BMJ has agreed a CC-BY licence shall apply, and/or iii) in accordance with the terms applicable for US Federal Government officers or employees acting as part of their official duties; on a worldwide, perpetual, irrevocable, royalty-free basis to BMJ Publishing Group Ltd ("BMJ") its licensees and where the relevant Journal is co-owned by BMJ to the co-owners of the Journal, to publish the Work in BMJ Open and any other BMJ products and to exploit all rights, as set out in our licence.

The Submitting Author accepts and understands that any supply made under these terms is made by BMJ to the Submitting Author unless you are acting as an employee on behalf of your employer or a postgraduate student of an affiliated institution which is paying any applicable article publishing charge ("APC") for Open Access articles. Where the Submitting Author wishes to make the Work available on an Open Access basis (and intends to pay the relevant APC), the terms of reuse of such Open Access shall be governed by a Creative Commons licence – details of these licences and which Creative Commons licence will apply to this Work are set out in our licence referred to above.

For peer review only

REFERENCES

1. Multimorbidity: clinical assessment and management (NICE clinical guideline 56): National Insitute for Health and Care Excellence., 2016.
2. Zacho J, Tybjaerg-Hansen A, Nordestgaard BG. C-reactive protein and all-cause mortality--the Copenhagen City Heart Study. *Eur Heart J* 2010;31(13):1624-32. doi: 10.1093/eurheartj/ehq103 [published Online First: 2010/04/29]
3. Strandberg TE, Tilvis RS. C-reactive protein, cardiovascular risk factors, and mortality in a prospective study in the elderly. *Arteriosclerosis, thrombosis, and vascular biology* 2000;20(4):1057-60. [published Online First: 2000/04/15]
4. Barron E, Lara J, White M, et al. Blood-borne biomarkers of mortality risk: systematic review of cohort studies. *PloS one* 2015;10(6):e0127550. doi: 10.1371/journal.pone.0127550 [published Online First: 2015/06/04]
5. Kaptoge S, Di Angelantonio E, Lowe G, et al. C-reactive protein concentration and risk of coronary heart disease, stroke, and mortality: an individual participant meta-analysis. *Lancet* 2010;375(9709):132-40. doi: 10.1016/s0140-6736(09)61717-7
6. Viasus D, Del Rio-Pertuz G, Simonetti AF, et al. Biomarkers for predicting short-term mortality in community-acquired pneumonia: A systematic review and meta-analysis. *The Journal of infection* 2016;72(3):273-82. doi: 10.1016/j.jinf.2016.01.002 [published Online First: 2016/01/19]
7. Gradel KO, Thomsen RW, Lundbye-Christensen S, et al. Baseline C-reactive protein level as a predictor of mortality in bacteraemia patients: a population-based cohort study. *Clinical Microbiology and Infection* 2011;17(4):627-32. doi: https://doi.org/10.1111/j.1469-0691.2010.03284.x
8. Li WJ, Chen XM, Nie XY, et al. Cardiac troponin and C-reactive protein for predicting all-cause and cardiovascular mortality in patients with chronic kidney disease: a meta-analysis. *Clinics (Sao Paulo, Brazil)* 2015;70(4):301-11. doi: 10.6061/clinics/2015(04)14 [published Online First: 2015/05/29]
9. Watson J, Whiting P, Banks J, Pyne Y, Hamilton W. Added value and cascade effects of inflammatory marker tests in UK primary care: a cohort study from the Clinical Practice Research Datalink. *BJGP* 2019; 69 (684): e470-e478. DOI: https://doi.org/10.3399/bjgp19X704321
10. Watson J, Salisbury C, Whiting P, Banks J, Hamilton W. Use of multiple inflammatory marker tests in primary care: using Clinical Practice Research Datalink to evaluate accuracy. *BJGP* 2019; 69 (684): e462-e469. DOI: https://doi.org/10.3399/bjgp19X704309
11. DeLong ER, DeLong DM, Clarke-Pearson DLJB. Comparing the areas under two or more correlated receiver operating characteristic curves: a nonparametric approach. 1988:837-45.
12. Stata Statistical Software: Release 15. [program]: College Station, TX: StataCorp LLC, 2017.
13. Watson J BS, Hamilton F, Hamilton W, Mounce L. Lessons from biases in electronic health record data: the importance of clinical vigilance with negative test results. *BMJ* 2018;361
14. Clegg A, Bates C, Young J, et al. Development and validation of an electronic frailty index using routine primary care electronic health record data. *Age and Ageing* 2016;45(3):353-60. doi: 10.1093/ageing/afw039 %J Age and Ageing
15. Hippisley-Cox J, Coupland C. Development and validation of QMortality risk prediction algorithm to estimate short term risk of death and assess frailty: cohort study. 2017;358:j4208. doi: 10.1136/bmj.j4208 %J BMJ
16. Deelen J, Kettunen J, Fischer K, et al. A metabolic profile of all-cause mortality risk identified in an observational study of 44,168 individuals. *Nature Communications* 2019;10(1):3346. doi: 10.1038/s41467-019-11311-9
17. Li Y, Zhong X, Cheng G, et al. Hs-CRP and all-cause, cardiovascular, and cancer mortality risk: a meta-analysis. *Atherosclerosis* 2017;259:75-82.

For peer review only

18. Ford ES, Cunningham TJ, Mannino DM. Inflammatory markers and mortality among US adults with obstructive lung function. *Respirology* 2015;20(4):587-93. doi: 10.1111/resp.12499 [published Online First: 2015/03/04]
19. Landman GW, Kleefstra N, Groenier KH, et al. Inflammation biomarkers and mortality prediction in patients with type 2 diabetes (ZODIAC-27). *Atherosclerosis* 2016;250:46-51. doi: 10.1016/j.atherosclerosis.2016.04.015 [published Online First: 2016/05/15]
20. Zhang W, He J, Zhang F, et al. Prognostic role of C-reactive protein and interleukin-6 in dialysis patients: a systematic review and meta-analysis. *Journal of nephrology* 2013;26(2):243-53. doi: 10.5301/jn.5000169 [published Online First: 2012/06/12]
21. Jing X, Huang C, Zhou H, et al. Association between serum C-reactive protein value and prognosis of patients with non-small cell lung cancer: a meta-analysis. *International journal of clinical and experimental medicine* 2015;8(7):10633-9. [published Online First: 2015/09/18]
22. Shrotriya S, Walsh D, Bennani-Baiti N, et al. C-Reactive Protein Is an Important Biomarker for Prognosis Tumor Recurrence and Treatment Response in Adult Solid Tumors: A Systematic Review. *PloS one* 2015;10(12):e0143080. doi: 10.1371/journal.pone.0143080 [published Online First: 2015/12/31]
23. Hamilton W, Round J. Identifying frailty in primary care. 2017;358:j4478. doi: 10.1136/bmj.j4478 %J BMJ
24. NHS England: Identifying frailty [Available from: https://www.england.nhs.uk/ourwork/clinical-policy/older-people/frailty/frailty-risk-identification/

For peer review only

Figure 1: Flowchart of one-year mortality (95% confidence intervals) according to CRP test results. The right-hand column shows one-year mortality according to repeat test result; defined as the first CRP test performed in the 3 months following the index date

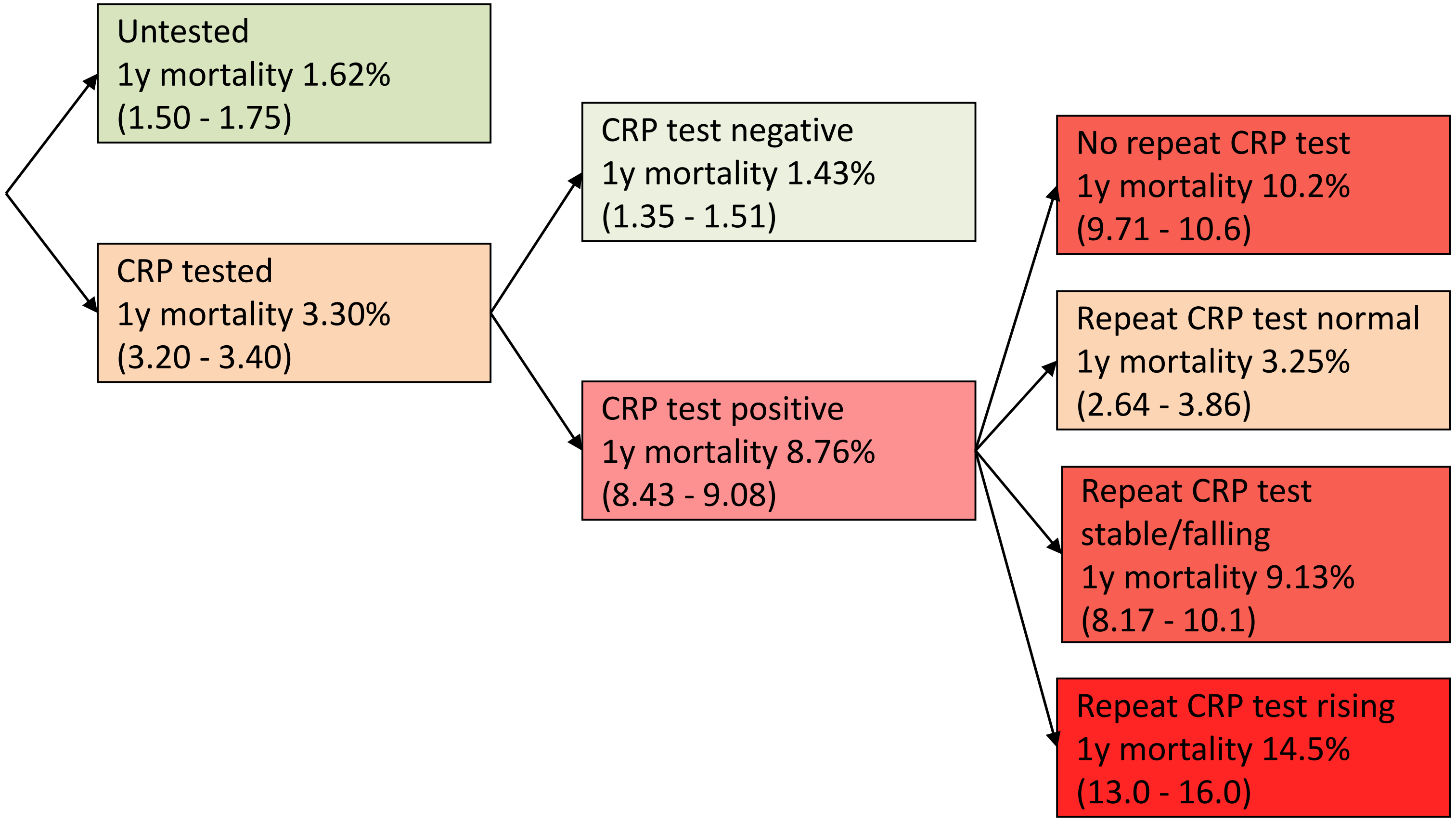

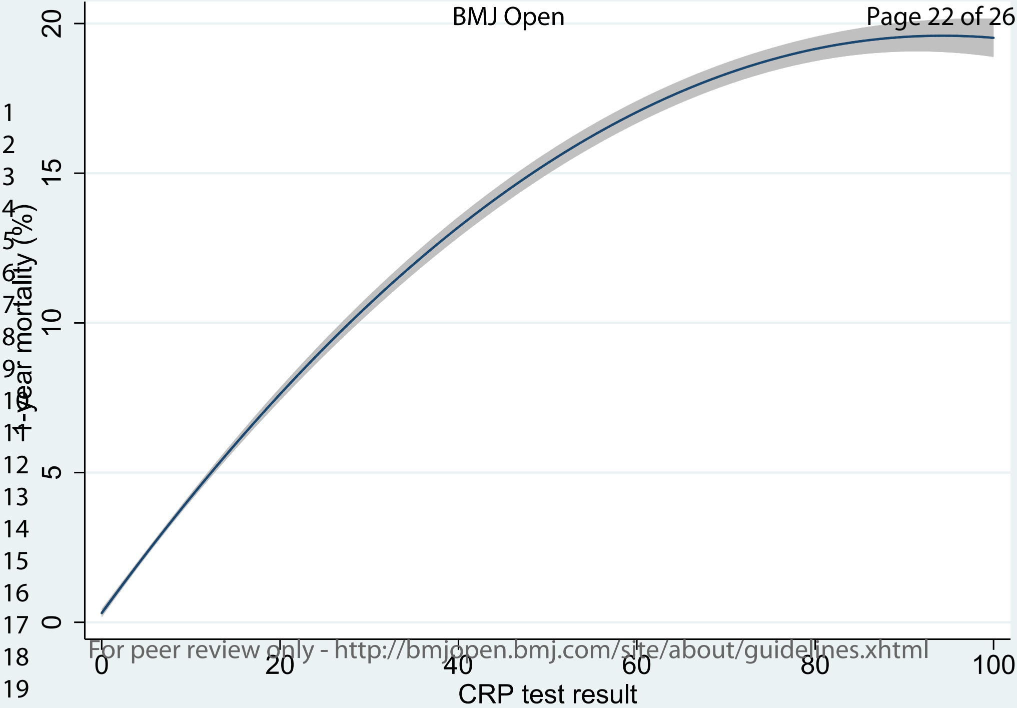
1-year mortality (%)
0
5
10
15
20
0
20
40
60
80
100
CRP test result

Supplementary Table 1: Characteristics of the tested and untested cohort, compared to the UK population

Patient Characteristic	Tested Cohort	Untested controls	National Population	GP Attendance
**Gender:**	**(159,325)**	**(39,928)**	*	**
Male	38.0%	38.2%	48.7%	39.8%
Female	62.0%	61.8%	51.3%	60.2%
**Age Group:**	**(159,325)**	**(39,928)**	*	**
18-29	10.9%	10.8%	20.2%	12.5%
30-39	11.4%	11.5%	16.4%	13.7%
40-49	15.9%	15.8%	17.8%	14.7%
50-59	17.6%	17.7%	16.3%	14.1%
60-69	17.7%	17.3%	13.9%	16.3%
70-79	15.1%	15.4%	9.4%	15.5%
≥80	11.5%	11.4%	6.0%	13.2%
**IMD Socioeconomic Status***:**	**(87,839)**	**(22,062)**		
1 (least deprived)	23.1%	23.9%	20.0%	
2	21.9%	22.2%	20.0%	
3	21.6%	21.4%	20.0%	
4	19.1%	19.0%	20.0%	
5 (most deprived)	14.2%	13.6%	20.0%	
**Ethnicity***:**	**(75,802)**	**(17,868)**	**	
White	87.8%	87.7%	85.4%	
Non-white	12.3%	12.4%	14.6%	

* Based on UK population estimates in mid-2014[1]
** Based on ONS – National Consultation Rates Report (2008)[2] (age 20-30 not 18 – 30)
*** IMD linkage and ethnicity data was not available for the complete dataset, hence a lower denominator for these figures shown

[1] Office for National Statistics. *Population Estimates for UK, England and Wales, Scotland and Northern Ireland, Mid-2014*. (2014).

[2] Hippisley-Cox, J. *ONS Consultations Report 20 - QRESEARCH calendar year consultations 2008*. (2008).

Supplementary table 2: Cause of death amongst patients with one or more raised inflammatory markers at the index date subdivided by age category (n=1,872 deaths)

Age group	Cause of death n (%)			
	Cancer	Cardiovascular	Respiratory	Other
<30	0 (0)	0 (0)	0 (0)	1 (100)
30-39	4 (40.0)	1 (10.0)	0 (0)	5 (50.0)
40-49	21 (45.7)	4 (8.7)	1 (2.2)	20 (43.5)
50-59	56 (56.6)	16 (16.2)	9 (9.1)	18 (18.2)
60-69	152 (61.0)	31 (12.5)	25 (10.0)	41 (16.5)
70-79	199 (46.6)	88 (20.6)	59 (13.8)	81 (19.0)
≥80	264 (25.4)	309 (29.7)	170 (16.4)	297 (28.6)
Total	696 (37.2)	449 (24.0)	264 (14.1)	463 (24.7)

For peer review only

Section & Topic	No	Item	Reported on page #
**TITLE OR ABSTRACT**			
	1	Identification as a study of diagnostic accuracy using at least one measure of accuracy (such as sensitivity, specificity, predictive values, or AUC)	Abstract page 2-3
**ABSTRACT**			
	2	Structured summary of study design, methods, results, and conclusions (for specific guidance, see STARD for Abstracts)	Abstract Page 2-3
**INTRODUCTION**			
	3	Scientific and clinical background, including the intended use and clinical role of the index test	5
	4	Study objectives and hypotheses	5
**METHODS**			
Study design	5	Whether data collection was planned before the index test and reference standard were performed (prospective study) or after (retrospective study)	6
Participants	6	Eligibility criteria	5-6
	7	On what basis potentially eligible participants were identified (such as symptoms, results from previous tests, inclusion in registry)	5-6
	8	Where and when potentially eligible participants were identified (setting, location and dates)	5-6
	9	Whether participants formed a consecutive, random or convenience series	5-6
Test methods	10a	Index test, in sufficient detail to allow replication	6
	10b	Reference standard, in sufficient detail to allow replication	6
	11	Rationale for choosing the reference standard (if alternatives exist)	n/a
	12a	Definition of and rationale for test positivity cut-offs or result categories of the index test, distinguishing pre-specified from exploratory	6
	12b	Definition of and rationale for test positivity cut-offs or result categories of the reference standard, distinguishing pre-specified from exploratory	n/a
	13a	Whether clinical information and reference standard results were available to the performers/readers of the index test	n/a
	13b	Whether clinical information and index test results were available to the assessors of the reference standard	n/a
Analysis	14	Methods for estimating or comparing measures of diagnostic accuracy	6
	15	How indeterminate index test or reference standard results were handled	n/a
	16	How missing data on the index test and reference standard were handled	6
	17	Any analyses of variability in diagnostic accuracy, distinguishing pre-specified from exploratory	
	18	Intended sample size and how it was determined	Sample size calculations are provided in linked paper which is referenced
**RESULTS**			
Participants	19	Flow of participants, using a diagram	In linked paper
	20	Baseline demographic and clinical characteristics of participants	Table 1 page 7
	21a	Distribution of severity of disease in those with the target condition	n/a (target condition = death)
	21b	Distribution of alternative diagnoses in those without the target condition	n/a
	22	Time interval and any clinical interventions between index test and reference standard	1 year mortality
Test results	23	Cross tabulation of the index test results (or their distribution) by the results of the reference standard	
	24	Estimates of diagnostic accuracy and their precision (such as 95% confidence intervals)	Table 2
	25	Any adverse events from performing the index test or the reference standard	n/a - observational
**DISCUSSION**			
	26	Study limitations, including sources of potential bias, statistical uncertainty, and generalisability	9
	27	Implications for practice, including the intended use and clinical role of the index test	10 - 11

For peer review only - http://bmjopen.bmj.com/site/about/guidelines.xhtml

OTHER INFORMATION			
	**28**	Registration number and name of registry	15
	**29**	Where the full study protocol can be accessed	15
	**30**	Sources of funding and other support; role of funders	15

For peer review only

STARD 2015

AIM

STARD stands for "Standards for Reporting Diagnostic accuracy studies". This list of items was developed to contribute to the completeness and transparency of reporting of diagnostic accuracy studies. Authors can use the list to write informative study reports. Editors and peer-reviewers can use it to evaluate whether the information has been included in manuscripts submitted for publication.

EXPLANATION

A **diagnostic accuracy study** evaluates the ability of one or more medical tests to correctly classify study participants as having a **target condition.** This can be a disease, a disease stage, response or benefit from therapy, or an event or condition in the future. A medical test can be an imaging procedure, a laboratory test, elements from history and physical examination, a combination of these, or any other method for collecting information about the current health status of a patient.

The test whose accuracy is evaluated is called **index test.** A study can evaluate the accuracy of one or more index tests. Evaluating the ability of a medical test to correctly classify patients is typically done by comparing the distribution of the index test results with those of the **reference standard**. The reference standard is the best available method for establishing the presence or absence of the target condition. An accuracy study can rely on one or more reference standards.

If test results are categorized as either positive or negative, the cross tabulation of the index test results against those of the reference standard can be used to estimate the **sensitivity** of the index test (the proportion of participants *with* the target condition who have a positive index test), and its **specificity** (the proportion *without* the target condition who have a negative index test). From this cross tabulation (sometimes referred to as the contingency or "2x2" table), several other accuracy statistics can be estimated, such as the positive and negative **predictive values** of the test. Confidence intervals around estimates of accuracy can then be calculated to quantify the statistical **precision** of the measurements.

If the index test results can take more than two values, categorization of test results as positive or negative requires a **test positivity cut-off**. When multiple such cut-offs can be defined, authors can report a receiver operating characteristic (ROC) curve which graphically represents the combination of sensitivity and specificity for each possible test positivity cut-off. The **area under the ROC curve** informs in a single numerical value about the overall diagnostic accuracy of the index test.

The **intended use** of a medical test can be diagnosis, screening, staging, monitoring, surveillance, prediction or prognosis. The **clinical role** of a test explains its position relative to existing tests in the clinical pathway. A replacement test, for example, replaces an existing test. A triage test is used before an existing test; an add-on test is used after an existing test.

Besides diagnostic accuracy, several other outcomes and statistics may be relevant in the evaluation of medical tests. Medical tests can also be used to classify patients for purposes other than diagnosis, such as staging or prognosis. The STARD list was not explicitly developed for these other outcomes, statistics, and study types, although most STARD items would still apply.

DEVELOPMENT

This STARD list was released in 2015. The 30 items were identified by an international expert group of methodologists, researchers, and editors. The guiding principle in the development of STARD was to select items that, when reported, would help readers to judge the potential for bias in the study, to appraise the applicability of the study findings and the validity of conclusions and recommendations. The list represents an update of the first version, which was published in 2003.

More information can be found on http://www.equator-network.org/reporting-guidelines/stard.

CC BY SA

For peer review only - http://bmjopen.bmj.com/site/about/guidelines.xhtml

BMJ Open

Raised inflammatory markers as a predictor of one-year mortality: A cohort study in primary care in the United Kingdom using electronic health record data

Journal:	*BMJ Open*
Manuscript ID	bmjopen-2019-036027.R2
Article Type:	Original research
Date Submitted by the Author:	07-Apr-2020
Complete List of Authors:	Watson, Jessica; University of Bristol, Centre for Academic Primary Care<br>Whiting, Penny; University of Bristol, School of Social and Community Medicine<br>Salisbury, Chris; University of Bristol, Academic Unit of Primary Health Care<br>Banks, Jonathan; University of Bristol, Social & Community Medicine<br>Hamilton, Willie; University of Exeter Medical School, Primary Care Diagnostics
**Primary Subject Heading**:	General practice / Family practice
Secondary Subject Heading:	Epidemiology, Geriatric medicine
Keywords:	PRIMARY CARE, EPIDEMIOLOGY, Chemical pathology < PATHOLOGY, GERIATRIC MEDICINE

SCHOLARONE™
Manuscripts

I, the Submitting Author has the right to grant and does grant on behalf of all authors of the Work (as defined in the below author licence), an exclusive licence and/or a non-exclusive licence for contributions from authors who are: i) UK Crown employees; ii) where BMJ has agreed a CC-BY licence shall apply, and/or iii) in accordance with the terms applicable for US Federal Government officers or employees acting as part of their official duties; on a worldwide, perpetual, irrevocable, royalty-free basis to BMJ Publishing Group Ltd ("BMJ") its licensees and where the relevant Journal is co-owned by BMJ to the co-owners of the Journal, to publish the Work in this journal and any other BMJ products and to exploit all rights, as set out in our licence.

The Submitting Author accepts and understands that any supply made under these terms is made by BMJ to the Submitting Author unless you are acting as an employee on behalf of your employer or a postgraduate student of an affiliated institution which is paying any applicable article publishing charge ("APC") for Open Access articles. Where the Submitting Author wishes to make the Work available on an Open Access basis (and intends to pay the relevant APC), the terms of reuse of such Open Access shall be governed by a Creative Commons licence – details of these licences and which Creative Commons licence will apply to this Work are set out in our licence referred to above.

Other than as permitted in any relevant BMJ Author's Self Archiving Policies, I confirm this Work has not been accepted for publication elsewhere, is not being considered for publication elsewhere and does not duplicate material already published. I confirm all authors consent to publication of this Work and authorise the granting of this licence.

For peer review only

Raised inflammatory markers as a predictor of one-year mortality: A cohort study in primary care in the United Kingdom using electronic health record data

Jessica Watson[1,2], Penny Whiting[1,2], Chris Salisbury[1,2], Jonathan Banks[1,2], Willie Hamilton[3]

1. Centre for Academic Primary Care, Department of Population Health Sciences, Bristol Medical School, University of Bristol, Bristol, UK
2. NIHR CLAHRC West, Bristol, UK
3. University of Exeter Medical School, Exeter, UK

Corresponding author:

Dr Jessica Watson

Centre for Academic Primary Care, Department of Population Health Sciences

Canynge Hall

39 Whatley Road

Bristol, BS8 2PS

Jessica.Watson@bristol.ac.uk

Tel: (+44) 07837002664

Word count: 2,094 (excluding abstract, references, figures and tables)

For peer review only

Abstract

Objectives

Identification of patients at increased mortality risk is important in the context of increasing multimorbidity and an ageing population, to help facilitate the planning and delivery of services. The aim of this study was to examine one-year all-cause mortality in a cohort of primary care patients in whom inflammatory markers including C-reactive protein (CRP), erythrocyte sedimentation rate (ESR) and plasma viscosity (PV), had been tested.

Design

Observational cohort study using GP Electronic Health Records from the Clinical Practice Research Datalink, with linkage to ONS Death Registry.

Setting

UK Primary Care

Participants

159,325 patients with inflammatory marker tests done in 2014 and 39,928 age, sex and practice matched controls without inflammatory marker testing. ONS Death registry data was available for 109,966 participants.

Primary and secondary outcome measures

One-year mortality in those with raised inflammatory markers compared to normal inflammatory markers and untested controls. Sub-analyses stratified one-year mortality by age-group, gender and cause of death.

For peer review only

Results

Patients with a raised inflammatory marker (n=47,797) had an overall one-year all-cause mortality of 6.89%, compared to 1.41% in those with normal inflammatory markers ($p<0.001$) and 1.62% in untested controls. A raised CRP is associated with the highest mortality rate at 8.76% compared to 4.99% for ESR and 4.66% for PV. One-year mortality is higher in men with a raised inflammatory marker compared to women (9.78% versus 5.29%). The c-statistic of a simple mortality prediction model containing age, sex and CRP test result is 0.89.

Conclusions

Inflammatory markers are a strong predictor of all-cause mortality in primary care, with a c-statistic comparable to several previously developed frailty indices. Future research should consider the added value of CRP testing, in combination with other risk factors, to improve prediction of mortality in primary care. Evidence based interventions for frailty are needed alongside predictive tools.

For peer review only

Strengths and limitations of this study

- The main strength of this study is it’s size and setting in primary care, making results relevant to clinical practice
- As test results were transferred electronically to the GP record, there is a very low risk of transcription error or bias
- Use of ONS death registry data increased the accuracy of recording of mortality, the primary outcome
- Main limitation is the lack of information on the reasons for testing
- Previous studies have focussed on predictors of mortality in the elderly, yet we were able to study mortality across all age groups

For peer review only

Background

Identification of patients at increased risk of mortality is important in the context of increasing multimorbidity and an ageing population, with the aim of helping facilitate the planning and delivery of services. Multiple risk tools have therefore been developed to predict mortality; these can be used to help predict frailty, unplanned hospital admissions and to allow targeted interventions to people at an increased mortality risk. The National Institute for Health and Care Excellence (NICE) multimorbidity guidelines systematically reviewed 41 of these mortality risk tools; the majority were of low or very low quality and a need for further research in this area was identified.[1] Current risk tools include variables such as disease status, sociodemographic factors and laboratory test results (eg anaemia, raised platelets), however, none in current use include an inflammatory marker test.

Inflammatory markers such as C reactive protein (CRP), erythrocyte sedimentation rate (ESR) and plasma viscosity (PV) are commonly used in primary care to aid diagnosis and monitoring of infections and inflammatory conditions. Cohort studies in the general population have reported inflammatory markers as predicting future mortality,[2-4] particularly from cardiovascular disease.[5] CRP has also been shown to have predictive value for mortality in hospital settings.[6-8] The clinical relevance of these findings in primary care settings, and over the shorter term, has not previously been described.

The aim of this study was to examine one-year all-cause mortality in a cohort of primary care patients in whom inflammatory marker bloods had been tested.

Methods

This was a secondary analysis of an observational cohort study of 160,000 patients aged >18 from the Clinical Practice Research Datalink (CPRD) with inflammatory marker blood testing in 2014, and

For peer review only

40,000 age, sex and practice matched controls without inflammatory marker testing also in 2014. The methods have been described fully in previous paired papers describing the disease outcomes following inflammatory marker testing.[9 10] Patients were excluded from the analysis if the inflammatory marker test result was missing (n=673) or if results were so abnormal as to be considered spurious (n=2). Linkage to ONS death registry data was available for 109,966. The three inflammatory marker tests studied were C-reactive protein (CRP), erythrocyte sedimentation rate (ESR) and plasma viscosity (PV). We defined a raised inflammatory marker using the mean upper limit of normal for laboratories within our study. For CRP this was 6.8mg/l, for simplicity rounded to 7mg/l; for PV 1.72mPa.s. For ESR this mean upper limit of normal was rounded and stratified by gender and age.[9] When the same inflammatory marker was repeated on the same day (n=231), we retained the highest value. The index date was defined as the first date of inflammatory marker testing in 2014, with one-year mortality defined as death within one year of this index date. Date of death was defined as the earlier date of recorded death in either CPRD or ONS death registry. Cause of death was available from death certification data in 3,141 out of 5,512 deaths where ONS linkage was available.

Analysis

The primary analysis compared one-year mortality in those with raised versus normal inflammatory markers and compared to untested controls. Sub-analyses stratified one-year mortality by age group and gender and cause of death. For each of the three tests (CRP, ESR and PV), dichotomised test results were cross-classified with the reference standard one-year mortality, allowing sensitivity and specificity to be calculated. Logistic regression was used to calculate diagnostic odds ratios, with and without adjustment for age and gender.

Test results were also analysed on a continuous scale, using logistic regression to determine the dose response relationship between inflammatory marker test result and mortality. The area under receiver operator curve (AUC), otherwise known as the c-statistic, was calculated using a logistic

For peer review only

regression model using test result on a continuous scale, with log transformation due to the skewed nature of the data, with and without age and gender as covariates. Comparisons of the c-statistic were made using DeLong method.[11] All analyses were done using Stata15.[12]

Patient and public involvement

Patients were not involved in the design, conduct, reporting or dissemination of this research.

Results

Demographics of the tested cohort and untested controls, compared to the UK population are shown in **supplementary table 1**. After exclusions there were 159,325 tested patients of whom 114,198 (71.7%) had a CRP test, 92,325 (58.0%) an ESR test, and 15,994 (10%) a PV test; 62,789 (39.4%) had more than one inflammatory marker performed simultaneously, mostly CRP and ESR together (51,546). Overall 47,797 (30%) had one or more raised inflammatory marker on the index date. In total 5,512 patients died within one-year of the index date; 648 deaths in the untested group, 1,572 deaths in the normal inflammatory marker group and 3,292 deaths in the group with one or more raised inflammatory marker.

Overall mortality rates

Table 1 shows overall mortality rates subdivided by age, gender and test results. Patients with a raised inflammatory marker (n=47,797) had an overall one-year mortality of 6.89%, compared to 1.41% in those with normal inflammatory markers ($p<0.001$). In the untested comparison cohort, one-year mortality was 1.62%. The association between raised inflammatory markers and one-year mortality was seen in all age groups apart from the under 30-year olds. In older age groups the absolute increase in risk was considerable; a raised inflammatory marker in the over 80s was

For peer review only

associated with a one-year mortality of 21.8%, compared to 8.6% in the over 80s with normal inflammatory markers.

Men with a raised inflammatory marker had a significantly higher one-year mortality rate than women with a raised inflammatory marker (9.78% versus 5.29%). Patients with a raised CRP had a one-year mortality of 8.76% compared to 4.99% for those with raised ESR and 4.66% for raised PV.

In the 62,789 patients with more than one inflammatory marker performed simultaneously on the index date, one-year mortality was higher in the 9,029 patients with concordant raised values at 6.9%, compared to the 13,783 with discordant results (one raised, one normal) who had a one-year mortality of 2.8%. In the 39,977 patients with two simultaneous negative inflammatory markers one-year mortality was 0.85%.

For peer review only

Table 1: One-year mortality (%, 95% CI) subdivided by age, gender and test result

	Untested (n=39,928)	Normal inflammatory markers (n=111,528)	Any raised inflammatory marker (n=47,797)	Raised CRP (n=29,164)	Raised ESR (n=23,138)	Raised PV (n=4,568)
Overall (n=199,253)	1.62 (1.50-1.75)	1.41 (1.34-1.48)	6.89 (6.66-7.11)	8.76 (8.43-9.08)	4.99 (4.71-5.27)	4.66 (4.05-5.27)
Age						
<30 (n=21,732)	0.09 (0.02-0.18)	0.04 (0.01-0.08)	0.08 (0.00-0.17)	0.13 (0.00-0.27)	0.00 (0.00-0.00)	0.0 (0.00-0.00)
30-39 (n=22,718)	0.04 (0.00-0.10)	0.08 (0.03-0.13)	0.37 (0.19-5.47)	0.50 (0.23-0.77)	0.28 (0.06-0.51)	0.0 (0.00-0.00)
40-49 (n=31,588)	0.19 (0.08-0.30)	0.15 (0.09-0.20)	1.12 (0.86-1.39)	1.54 (1.14-1.95)	0.92 (0.59-1.25)	0.90 (0.01-1.69)
50-59 (n=35,044)	0.41 (0.26-0.56)	0.41 (0.32-0.49)	2.37 (2.03-2.70)	2.98 (2.48-3.47)	1.79 (1.38-2.21)	1.62 (0.75-2.50)
60-69 (n=35,094)	0.84 (0.62-1.05)	0.91 (0.78-1.05)	4.96 (4.51-5.40)	6.72 (6.05-7.39)	3.88 (3.33-4.43)	3.27 (2.12-4.42)
70-79 (30,251)	2.27 (1.90-2.64)	2.51 (2.26-2.76)	9.39 (8.77-10.0)	11.38 (10.5-12.2)	7.35 (6.54-8.16)	6.32 (4.75-7.88)
>80 (22,826)	8.88 (8.05-9.70)	8.61 (8.07-9.16)	21.8 (20.9-22.7)	25.9 (24.7-27.1)	16.1 (14.9-17.3)	15.6 (12.9-18.3)
Gender						
Male (n=75,787)	1.86 (1.64-2.07)	1.58 (1.46-1.70)	9.78 (9.33-10.2)	11.48 (10.9-12.1)	7.98 (7.35-8.61)	6.63 (5.44-7.81)
Female (n=123,466)	1.48 (1.33-1.63)	1.30 (1.22-1.39)	5.29 (5.04-5.54)	6.99 (6.61-7.36)	3.65 3.36-3.94)	3.51 (2.84-4.18)

Table 2 shows the performance characteristics of inflammatory markers, including sensitivity, specificity and c-statistic. CRP had the highest sensitivity of the three tests at 67.8% and the greatest c-statistic at 0.78. Odds ratios (OR) reduced after adjustment for age and gender but were still significant with an adjusted OR for a raised CRP of 4.5 ($p<0.001$), 2.9 for raised ESR and 2.1 for raised PV.

A logistic regression model containing age (as a continuous variable) and gender had a c-statistic of 0.85, compared to 0.89 for a full model containing age, gender and CRP test result as a continuous variable ($p < 0.001$); 0.88 with age, gender and ESR ($p < 0.001$); and 0.87 with age gender and PV ($p < 0.001$).

For peer review only

Table 2: Performance characteristics of CRP, ESR and PV for predicting one-year mortality

				**Univariable logistic regression**		**Adjusted for age and gender**	
	Sensitivity	Specificity	C-statistic*	OR	P	OR	P
**CRP**	67.8% (66.3-69.3)	75.9% (75.6-76.2)	0.78 (0.77-0.78)	6.6 (6.2-7.1)	<0.001	4.5 (4.2-4.8)	<0.001
**ESR**	56.6% (54.4-58.7)	75.7% (75.4-75.9)	0.66 (0.65-0.67	4.1 (3.7-4.4)	<0.001	2.9 (2.7-3.2)	<0.001
**PV**	52.0% (47.0-56.9)	72.1% (71.3-72.8)	0.62 (0.60-0.64)	2.8 (2.3-3.4)	<0.001	2.1 (1.7-2.6)	<0.001

*C-statistic calculated using log transformed test results as a continuous variable

Repeat testing

Figure 1 shows the one-year mortality in patients according to the subsequent repeat inflammatory marker results, using the most common test performed; CRP. The fact that a CRP test was requested by a GP was in itself, predictive of increased mortality, with one-year mortality of 3.3% in the tested versus 1.6% in the untested cohort. This increased to 8.76% one-year mortality if a single CRP test was raised, 9.13% if a second test was persistently raised and 14.5% if the second test was raised further still. Those with a raised inflammatory marker which was not subsequently rechecked had a one-year mortality rate of 10.2%, compared to 3.25% if a subsequent CRP normalised.

Figure 1: Flowchart of one-year mortality (95% confidence intervals) according to CRP test results. The right-hand column shows one-year mortality according to repeat test result; defined as the first CRP test performed in the 3 months following the index date

Dose response relationship

A dose response relationship was found between result of the index CRP test as a continuous variable and one-year mortality (figure 2). In 2,184 people with a CRP >=100mg/L overall one-year

For peer review only

mortality was 20.2%. Similar associations, with wider confidence intervals, were found for ESR and PV (not shown).

Figure 2: Polynomial logistic regression of mortality against CRP test result as a continuous variable

Cause of death

Cause of death from ONS death certification was available for 3,141 out of 5,512 total deaths in the cohort. **Table 3** summarises the cause of death amongst patients with raised inflammatory markers, compared to those with normal inflammatory markers, and untested controls. The commonest cause of death in the 26,507 patients with raised inflammatory markers was cancer (696 deaths) followed by cardiovascular disease (449 deaths). Odds of mortality in the raised versus normal inflammatory marker groups was highest for cancer (adjusted OR 6.34), followed by infections (adjusted OR 4.11). However significant increased odds of mortality were seen for all disease categories with the exception of deaths due to falls, musculoskeletal causes and senility. **Supplementary table 2** shows cause of death by age group for patients with raised inflammatory markers; cancer was the commonest cause of death in 40-79 year olds, cardiovascular disease increased with age and was the commonest cause of death in the over 80 age group.

For peer review only

Table 3: Cause of death amongst patients with ONS death registry linkage (n=109,966)

Cause of death	Untested controls (n=22,069)		Normal inflammatory markers (n=61,390)		Raised inflammatory markers (n=26,507)		Comparison between normal and raised inflammatory markers	
	Number of deaths	1 year mortality (%)	Number of deaths	1 year mortality (%)	Number of deaths	1 year mortality (%)	Unadjusted odds ratio (CI)	Odds ratio, adjusted for age and gender (CI)
All-cause mortality	380	1.74	889	1.45	1,872	7.08	5.16* (4.76-5.60)	3.66* (3.37-3.99)
Cancer	86	0.39	195	0.32	696	2.63	8.46* (7.21-9.93)	6.34* (5.40-7.46)
Cardiovascular disease	115	0.52	295	0.48	449	1.69	3.57* (3.08-4.14)	2.18* (1.87- 2.54)
Respiratory	53	0.24	141	0.23	264	1.00	4.37* (3.56-5.36)	2.68* (2.18-3.30)
Dementia	38	0.17	69	0.11	119	0.45	4.01* (2.98-5.39)	2.21* (1.64-2.99)
Gastrointestinal	16	0.07	42	0.07	86	0.32	4.75* (3.29-6.88)	3.58* (2.46-5.20)
Genitourinary	8	0.04	16	0.03	39	0.15	5.65* (3.16-10.1)	3.13* (1.74-5.64)
Infection	5	0.02	11	0.02	29	0.11	6.11* (3.05-12.2)	4.08* (2.03-8.23)
Blood disorder	2	0.01	11	0.02	25	0.09	5.27* (2.59-10.7)	3.11 (1.52-6.37)
Senility	12	0.05	8	0.01	21	0.08	6.08* (2.69-13.7)	2.80 (1.23-6.38)
Musculoskeletal	3	0.01	8	0.01	14	0.05	4.05* (1.70-9.67)	2.40 (1.00-5.77)
Falls	4	0.02	10	0.02	6	0.02	1.39 (0.51-3.82)	0.80 (0.29-2.22)
Other	38	0.17	83	0.14	124	0.47	3.47* (2.62-4.59)	2.41* (1.82-3.20)

*p<0.001

Discussion

Inflammatory markers are a strong predictor of all-cause mortality in primary care. The association between raised inflammatory markers and all-cause mortality is seen in all age groups except patients aged less than thirty years. Men with raised inflammatory markers have a higher one-year mortality than women (9.78% vs 5.29%). Of the three tests examined, CRP has the highest predictive accuracy for mortality. The overall c-statistic of a model containing age, sex and CRP test result of

For peer review only

0.89 is comparable to several previously developed frailty indices. Inflammatory markers could potentially be a simple indicator to improve prediction of life-expectancy in primary care.

Strengths and limitations

The major strength of this study is its size and its setting in primary care, making results relevant to clinical practice. As test results were transferred electronically to the GP record, there is a very low risk of transcription error or bias. Use of ONS death registry data increased the accuracy of recording of mortality, the primary outcome. Previous studies have focussed on predictors of mortality in the elderly, yet we were able to study mortality across all age groups. The fact that mortality is *not* raised in patients with a normal inflammatory marker suggests that the test result, rather than the clinician's decision to test, is significant.[13]

The main weakness is the lack of information about the reasons for testing; we cannot determine which tests were done for diagnosis, monitoring or non-specific purposes. However, this is also a strength, as it increases the generalisability of the results, which are not limited to specific subgroups of tested patients.

Comparison to previous literature

Several previous frailty indices have previously been developed, the most commonly used being the electronic frailty index (eFI)[14] and Qmortality.[15] The former has a c-statistic of 0.76; the latter a c-statistic of 0.85 for women and 0.84 for men. More recent research by Deelen *et al* has used combinations of biomarkers to predict mortality across all age groups; 226 potential biomarkers were selected, but CRP, ESR and PV were not considered.[16] They generated a model using 14 biomarkers with a c-statistic of 0.837: however, of the biomarkers considered, only albumin is

available in primary care, limiting the clinical usefulness of their findings. CRP by comparison is a low cost and widely available test. With a c-statistic of 0.78 for CRP alone, and 0.89 for a model including CRP, age and gender, inflammatory markers could be a simple indicator with a comparable accuracy to currently used mortality prediction tools.

The association between CRP and mortality is in keeping with population-based studies examining all-cause mortality[2 17] and cardiovascular mortality,[5] as well as hospital-based studies of patients with specific diseases including COPD,[18] diabetes,[19] chronic kidney disease,[8 20] pneumonia[6] and cancer.[21 22] The research reported here demonstrates that this association is also seen in a primary care setting, and over the shorter term. The finding that men with raised inflammatory markers are at higher mortality risk than women may reflect gender differences in healthcare-seeking behaviour in primary care; men have lower rates of consultation, so might be 'sicker' on average when presenting for blood tests.

Previous research has shown limited diagnostic utility of inflammatory markers in a primary care setting, where sensitivity is low, false positives are common, and abnormal tests can lead to increased rates of GP consultations, tests and referrals.[9] The fact that inflammatory markers have a higher c-statistic for mortality than for cancer, infections or autoimmune diseases,[10] may reflect the fact that inflammatory markers have both diagnostic and prognostic utility for a broad range of pathologies.

Clinical implications

General practitioners should interpret raised inflammatory markers within the wider clinical context; where the cause of inflammation is identifiable and treatable, mortality risks should not cause undue alarm. The findings back up the current clinical practice of repeating an abnormal test; a subsequent normal result is reassuring with mortality risk reducing to near normal. However,

For peer review only

clinicians should consider whether older patients with a persistently raised inflammatory markers are reaching the end of life.

There is debate over the utility of predicting mortality,[23] given the lack of evidence-based interventions. We would not recommend that clinicians test inflammatory markers purely for the purpose of mortality prediction, particularly given that false positives have been shown to lead to cascades of follow on tests, appointments and referrals.[9] However, general practitioners are already required to identify patients who are frail,[24] and inflammatory marker tests are commonly performed for many other reasons. Inflammatory marker test results, when available, may therefore add useful information to improve prediction of mortality and assessment of frailty in primary care.

Unanswered questions and future research

Future research should consider the added value of CRP testing in combination with other risk factors, to improve prediction of mortality in primary care. Predicting mortality in itself, however, is not enough, as tests will only benefit patients if they influence management. Evidence based interventions for frailty must therefore be developed alongside predictive tools.

For peer review only

Footnotes

Patient consent for publication:

Not required

Contributors:

WH, CS, PW, JB and JW all contributed to conception, design and planning of the study. JW analysed the data as part of her Doctoral Research Fellowship, with oversight of the conduct of the study provided by her PhD supervisors WH, CS, PW and JB. JW wrote the first draft of the paper. WH, CS, PW, JB and JW all contributed to subsequent drafts and read and approved the final manuscript.

Funding:

This report is independent research arising from JW's Doctoral Research Fellowship (DRF-2016-09-034) supported by the National Institute for Health Research.

JB, PW, CS and JW were supported by the National Institute for Health Research Applied Research Collaboration West (NIHR ARC West). CS is an NIHR Senior Investigator. The views expressed in this publication are those of the authors and not necessarily those of the NHS, the National Institute for Health Research, Health Education England or the Department of Health and Social Care.

Competing interests:

None declared

Data sharing statement:

This study is based on data from the Clinical Practice Research Datalink (CPRD) obtained under licence from the UK Medicines and Healthcare products Regulatory Agency, which does not permit data sharing. The data is provided by patients and collected by the NHS as part of their care and support. The interpretation and conclusions contained in this study are those of the authors alone.

Ethical approval

This study was approved by the Independent Scientific Application Committee for MHRA database research (ISAC) protocol number 17_003. The protocol is available at: https://www.cprd.com/protocol/diagnostic-utility-inflammatory-markers-primary-care-prospective-cohort-study

For peer review only

I, the Submitting Author has the right to grant and does grant on behalf of all authors of the Work (as defined in the below author licence), an exclusive licence and/or a non-exclusive licence for contributions from authors who are: i) UK Crown employees; ii) where BMJ has agreed a CC-BY licence shall apply, and/or iii) in accordance with the terms applicable for US Federal Government officers or employees acting as part of their official duties; on a worldwide, perpetual, irrevocable, royalty-free basis to BMJ Publishing Group Ltd ("BMJ") its licensees and where the relevant Journal is co-owned by BMJ to the co-owners of the Journal, to publish the Work in BMJ Open and any other BMJ products and to exploit all rights, as set out in our licence.

The Submitting Author accepts and understands that any supply made under these terms is made by BMJ to the Submitting Author unless you are acting as an employee on behalf of your employer or a postgraduate student of an affiliated institution which is paying any applicable article publishing charge ("APC") for Open Access articles. Where the Submitting Author wishes to make the Work available on an Open Access basis (and intends to pay the relevant APC), the terms of reuse of such Open Access shall be governed by a Creative Commons licence – details of these licences and which Creative Commons licence will apply to this Work are set out in our licence referred to above.

For peer review only

REFERENCES

1. Multimorbidity: clinical assessment and management (NICE clinical guideline 56): National Insitute for Health and Care Excellence., 2016.
2. Zacho J, Tybjaerg-Hansen A, Nordestgaard BG. C-reactive protein and all-cause mortality--the Copenhagen City Heart Study. *Eur Heart J* 2010;31(13):1624-32. doi: 10.1093/eurheartj/ehq103 [published Online First: 2010/04/29]
3. Strandberg TE, Tilvis RS. C-reactive protein, cardiovascular risk factors, and mortality in a prospective study in the elderly. *Arteriosclerosis, thrombosis, and vascular biology* 2000;20(4):1057-60. [published Online First: 2000/04/15]
4. Barron E, Lara J, White M, et al. Blood-borne biomarkers of mortality risk: systematic review of cohort studies. *PloS one* 2015;10(6):e0127550. doi: 10.1371/journal.pone.0127550 [published Online First: 2015/06/04]
5. Kaptoge S, Di Angelantonio E, Lowe G, et al. C-reactive protein concentration and risk of coronary heart disease, stroke, and mortality: an individual participant meta-analysis. *Lancet* 2010;375(9709):132-40. doi: 10.1016/s0140-6736(09)61717-7
6. Viasus D, Del Rio-Pertuz G, Simonetti AF, et al. Biomarkers for predicting short-term mortality in community-acquired pneumonia: A systematic review and meta-analysis. *The Journal of infection* 2016;72(3):273-82. doi: 10.1016/j.jinf.2016.01.002 [published Online First: 2016/01/19]
7. Gradel KO, Thomsen RW, Lundbye-Christensen S, et al. Baseline C-reactive protein level as a predictor of mortality in bacteraemia patients: a population-based cohort study. *Clinical Microbiology and Infection* 2011;17(4):627-32. doi: https://doi.org/10.1111/j.1469-0691.2010.03284.x
8. Li WJ, Chen XM, Nie XY, et al. Cardiac troponin and C-reactive protein for predicting all-cause and cardiovascular mortality in patients with chronic kidney disease: a meta-analysis. *Clinics (Sao Paulo, Brazil)* 2015;70(4):301-11. doi: 10.6061/clinics/2015(04)14 [published Online First: 2015/05/29]
9. Watson J, Whiting P, Banks J, Pyne Y, Hamilton W. Added value and cascade effects of inflammatory marker tests in UK primary care: a cohort study from the Clinical Practice Research Datalink. *BJGP* 2019; 69 (684): e470-e478. DOI: https://doi.org/10.3399/bjgp19X704321
10. Watson J, Salisbury C, Whiting P, Banks J, Hamilton W. Use of multiple inflammatory marker tests in primary care: using Clinical Practice Research Datalink to evaluate accuracy. *BJGP* 2019; 69 (684): e462-e469. DOI: https://doi.org/10.3399/bjgp19X704309
11. DeLong ER, DeLong DM, Clarke-Pearson DLJB. Comparing the areas under two or more correlated receiver operating characteristic curves: a nonparametric approach. 1988:837-45.
12. Stata Statistical Software: Release 15. [program]: College Station, TX: StataCorp LLC, 2017.
13. Watson J BS, Hamilton F, Hamilton W, Mounce L. Lessons from biases in electronic health record data: the importance of clinical vigilance with negative test results. *BMJ* 2018;361
14. Clegg A, Bates C, Young J, et al. Development and validation of an electronic frailty index using routine primary care electronic health record data. *Age and Ageing* 2016;45(3):353-60. doi: 10.1093/ageing/afw039 %J Age and Ageing
15. Hippisley-Cox J, Coupland C. Development and validation of QMortality risk prediction algorithm to estimate short term risk of death and assess frailty: cohort study. 2017;358:j4208. doi: 10.1136/bmj.j4208 %J BMJ
16. Deelen J, Kettunen J, Fischer K, et al. A metabolic profile of all-cause mortality risk identified in an observational study of 44,168 individuals. *Nature Communications* 2019;10(1):3346. doi: 10.1038/s41467-019-11311-9
17. Li Y, Zhong X, Cheng G, et al. Hs-CRP and all-cause, cardiovascular, and cancer mortality risk: a meta-analysis. *Atherosclerosis* 2017;259:75-82.

For peer review only

18. Ford ES, Cunningham TJ, Mannino DM. Inflammatory markers and mortality among US adults with obstructive lung function. *Respirology* 2015;20(4):587-93. doi: 10.1111/resp.12499 [published Online First: 2015/03/04]
19. Landman GW, Kleefstra N, Groenier KH, et al. Inflammation biomarkers and mortality prediction in patients with type 2 diabetes (ZODIAC-27). *Atherosclerosis* 2016;250:46-51. doi: 10.1016/j.atherosclerosis.2016.04.015 [published Online First: 2016/05/15]
20. Zhang W, He J, Zhang F, et al. Prognostic role of C-reactive protein and interleukin-6 in dialysis patients: a systematic review and meta-analysis. *Journal of nephrology* 2013;26(2):243-53. doi: 10.5301/jn.5000169 [published Online First: 2012/06/12]
21. Jing X, Huang C, Zhou H, et al. Association between serum C-reactive protein value and prognosis of patients with non-small cell lung cancer: a meta-analysis. *International journal of clinical and experimental medicine* 2015;8(7):10633-9. [published Online First: 2015/09/18]
22. Shrotriya S, Walsh D, Bennani-Baiti N, et al. C-Reactive Protein Is an Important Biomarker for Prognosis Tumor Recurrence and Treatment Response in Adult Solid Tumors: A Systematic Review. *PloS one* 2015;10(12):e0143080. doi: 10.1371/journal.pone.0143080 [published Online First: 2015/12/31]
23. Hamilton W, Round J. Identifying frailty in primary care. 2017;358:j4478. doi: 10.1136/bmj.j4478 %J BMJ
24. NHS England: Identifying frailty [Available from: https://www.england.nhs.uk/ourwork/clinical-policy/older-people/frailty/frailty-risk-identification/

For peer review only

Figure 1: Flowchart of one-year mortality (95% confidence intervals) according to CRP test results. The right-hand column shows one-year mortality according to repeat test result; defined as the first CRP test performed in the 3 months following the index date

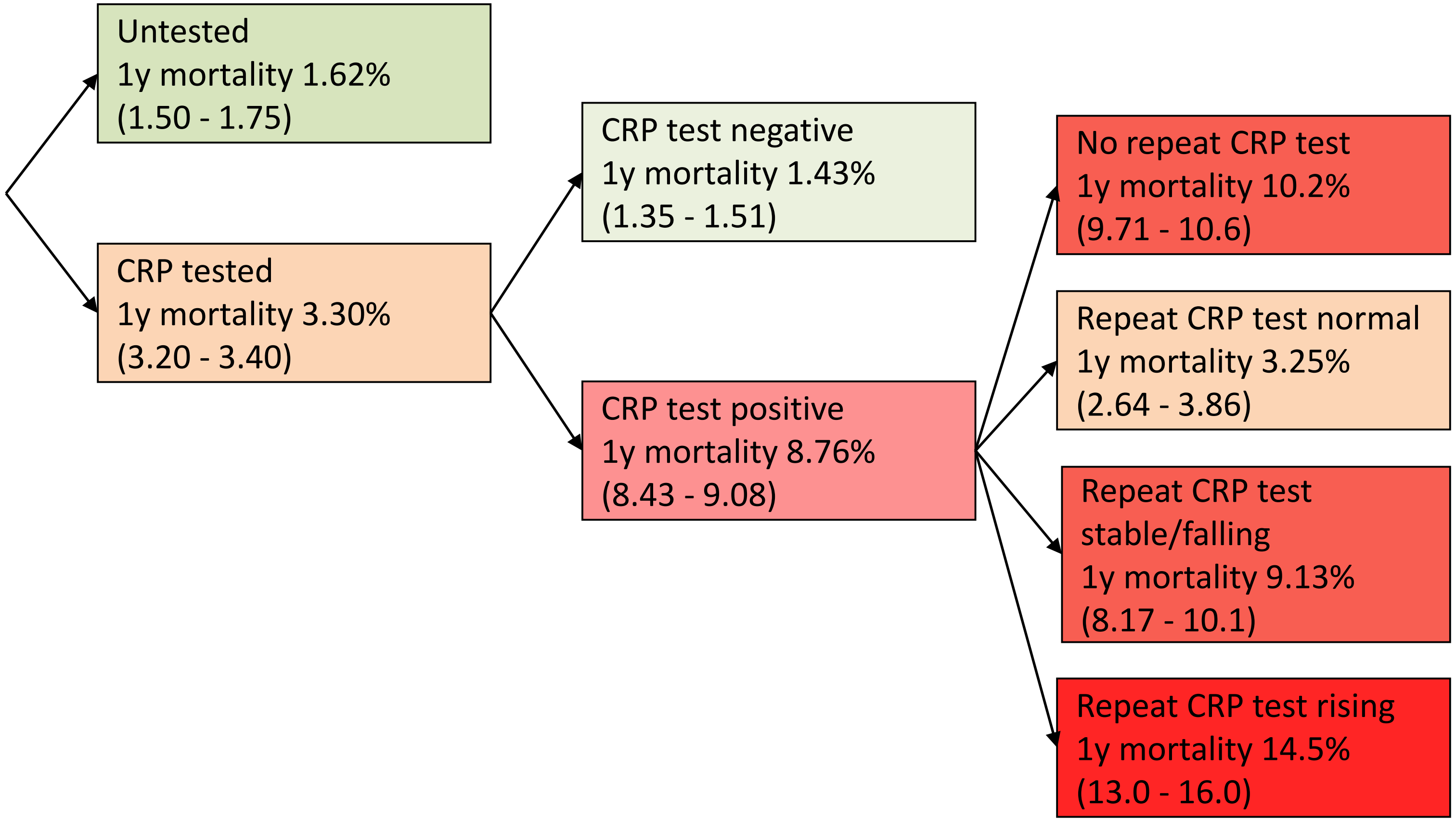

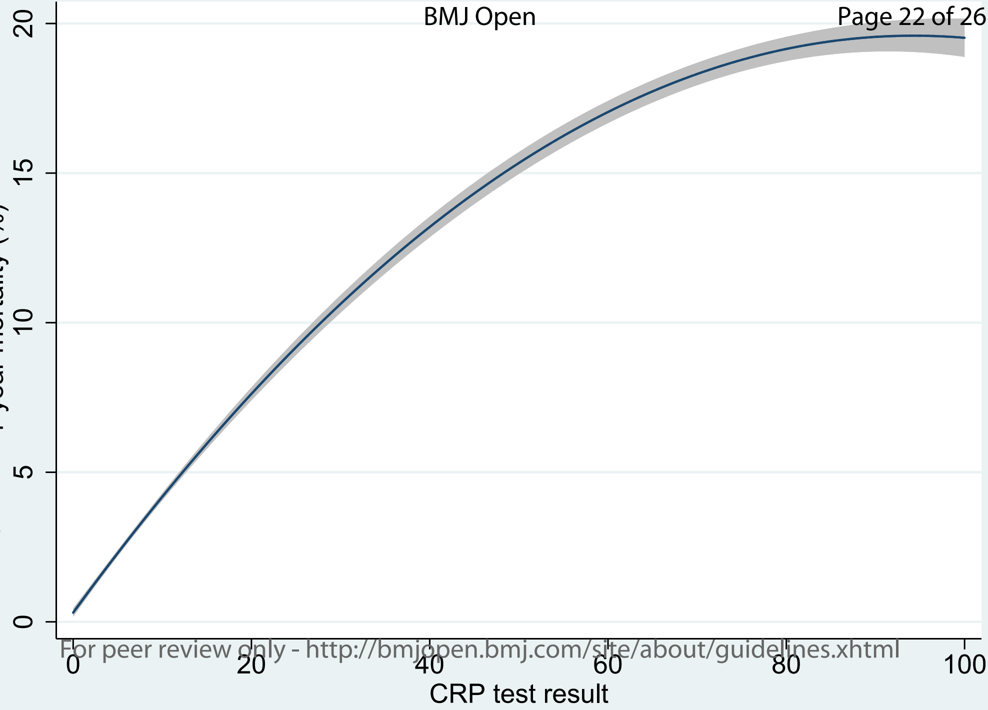

For peer review only - http://bmjopen.bmj.com/site/about/guidelines.xhtml

Supplementary Table 1: Characteristics of the tested and untested cohort, compared to the UK population

Patient Characteristic	Tested Cohort	Untested controls	National Population	GP Attendance
**Gender:**	**(159,325)**	**(39,928)**	*	**
Male	38.0%	38.2%	48.7%	39.8%
Female	62.0%	61.8%	51.3%	60.2%
**Age Group:**	**(159,325)**	**(39,928)**	*	**
18-29	10.9%	10.8%	20.2%	12.5%
30-39	11.4%	11.5%	16.4%	13.7%
40-49	15.9%	15.8%	17.8%	14.7%
50-59	17.6%	17.7%	16.3%	14.1%
60-69	17.7%	17.3%	13.9%	16.3%
70-79	15.1%	15.4%	9.4%	15.5%
≥80	11.5%	11.4%	6.0%	13.2%
**IMD Socioeconomic Status***:**	**(87,839)**	**(22,062)**		
1 (least deprived)	23.1%	23.9%	20.0%	
2	21.9%	22.2%	20.0%	
3	21.6%	21.4%	20.0%	
4	19.1%	19.0%	20.0%	
5 (most deprived)	14.2%	13.6%	20.0%	
**Ethnicity***:**	**(75,802)**	**(17,868)**	**	
White	87.8%	87.7%	85.4%	
Non-white	12.3%	12.4%	14.6%	

* Based on UK population estimates in mid-2014[1]
** Based on ONS – National Consultation Rates Report (2008)[2] (age 20-30 not 18 – 30)
*** IMD linkage and ethnicity data was not available for the complete dataset, hence a lower denominator for these figures shown

[1] Office for National Statistics. *Population Estimates for UK, England and Wales, Scotland and Northern Ireland, Mid-2014*. (2014).

[2] Hippisley-Cox, J. *ONS Consultations Report 20 - QRESEARCH calendar year consultations 2008*. (2008).

Supplementary table 2: Cause of death amongst patients with one or more raised inflammatory markers at the index date subdivided by age category (n=1,872 deaths)

Age group	Cause of death n (%)			
	Cancer	Cardiovascular	Respiratory	Other
<30	0 (0)	0 (0)	0 (0)	1 (100)
30-39	4 (40.0)	1 (10.0)	0 (0)	5 (50.0)
40-49	21 (45.7)	4 (8.7)	1 (2.2)	20 (43.5)
50-59	56 (56.6)	16 (16.2)	9 (9.1)	18 (18.2)
60-69	152 (61.0)	31 (12.5)	25 (10.0)	41 (16.5)
70-79	199 (46.6)	88 (20.6)	59 (13.8)	81 (19.0)
≥80	264 (25.4)	309 (29.7)	170 (16.4)	297 (28.6)
Total	696 (37.2)	449 (24.0)	264 (14.1)	463 (24.7)

For peer review only

Section & Topic	No	Item	Reported on page #
**TITLE OR ABSTRACT**			
	1	Identification as a study of diagnostic accuracy using at least one measure of accuracy (such as sensitivity, specificity, predictive values, or AUC)	Abstract page 2-3
**ABSTRACT**			
	2	Structured summary of study design, methods, results, and conclusions (for specific guidance, see STARD for Abstracts)	Abstract Page 2-3
**INTRODUCTION**			
	3	Scientific and clinical background, including the intended use and clinical role of the index test	5
	4	Study objectives and hypotheses	5
**METHODS**			
Study design	5	Whether data collection was planned before the index test and reference standard were performed (prospective study) or after (retrospective study)	6
Participants	6	Eligibility criteria	5-6
	7	On what basis potentially eligible participants were identified (such as symptoms, results from previous tests, inclusion in registry)	5-6
	8	Where and when potentially eligible participants were identified (setting, location and dates)	5-6
	9	Whether participants formed a consecutive, random or convenience series	5-6
Test methods	10a	Index test, in sufficient detail to allow replication	6
	10b	Reference standard, in sufficient detail to allow replication	6
	11	Rationale for choosing the reference standard (if alternatives exist)	n/a
	12a	Definition of and rationale for test positivity cut-offs or result categories of the index test, distinguishing pre-specified from exploratory	6
	12b	Definition of and rationale for test positivity cut-offs or result categories of the reference standard, distinguishing pre-specified from exploratory	n/a
	13a	Whether clinical information and reference standard results were available to the performers/readers of the index test	n/a
	13b	Whether clinical information and index test results were available to the assessors of the reference standard	n/a
Analysis	14	Methods for estimating or comparing measures of diagnostic accuracy	6
	15	How indeterminate index test or reference standard results were handled	n/a
	16	How missing data on the index test and reference standard were handled	6
	17	Any analyses of variability in diagnostic accuracy, distinguishing pre-specified from exploratory	
	18	Intended sample size and how it was determined	Sample size calculations are provided in linked paper which is referenced
**RESULTS**			
Participants	19	Flow of participants, using a diagram	In linked paper
	20	Baseline demographic and clinical characteristics of participants	Table 1 page 7
	21a	Distribution of severity of disease in those with the target condition	n/a (target condition = death)
	21b	Distribution of alternative diagnoses in those without the target condition	n/a
	22	Time interval and any clinical interventions between index test and reference standard	1 year mortality
Test results	23	Cross tabulation of the index test results (or their distribution) by the results of the reference standard	
	24	Estimates of diagnostic accuracy and their precision (such as 95% confidence intervals)	Table 2
	25	Any adverse events from performing the index test or the reference standard	n/a - observational
**DISCUSSION**			
	26	Study limitations, including sources of potential bias, statistical uncertainty, and generalisability	9
	27	Implications for practice, including the intended use and clinical role of the index test	10 - 11

cc BY SA

**OTHER INFORMATION**			
	**28**	Registration number and name of registry	15
	**29**	Where the full study protocol can be accessed	15
	**30**	Sources of funding and other support; role of funders	15

For peer review only

STARD 2015

AIM

STARD stands for “Standards for Reporting Diagnostic accuracy studies”. This list of items was developed to contribute to the completeness and transparency of reporting of diagnostic accuracy studies. Authors can use the list to write informative study reports. Editors and peer-reviewers can use it to evaluate whether the information has been included in manuscripts submitted for publication.

EXPLANATION

A **diagnostic accuracy study** evaluates the ability of one or more medical tests to correctly classify study participants as having a **target condition.** This can be a disease, a disease stage, response or benefit from therapy, or an event or condition in the future. A medical test can be an imaging procedure, a laboratory test, elements from history and physical examination, a combination of these, or any other method for collecting information about the current health status of a patient.

The test whose accuracy is evaluated is called **index test.** A study can evaluate the accuracy of one or more index tests. Evaluating the ability of a medical test to correctly classify patients is typically done by comparing the distribution of the index test results with those of the **reference standard**. The reference standard is the best available method for establishing the presence or absence of the target condition. An accuracy study can rely on one or more reference standards.

If test results are categorized as either positive or negative, the cross tabulation of the index test results against those of the reference standard can be used to estimate the **sensitivity** of the index test (the proportion of participants *with* the target condition who have a positive index test), and its **specificity** (the proportion *without* the target condition who have a negative index test). From this cross tabulation (sometimes referred to as the contingency or “2x2” table), several other accuracy statistics can be estimated, such as the positive and negative **predictive values** of the test. Confidence intervals around estimates of accuracy can then be calculated to quantify the statistical **precision** of the measurements.

If the index test results can take more than two values, categorization of test results as positive or negative requires a **test positivity cut-off**. When multiple such cut-offs can be defined, authors can report a receiver operating characteristic (ROC) curve which graphically represents the combination of sensitivity and specificity for each possible test positivity cut-off. The **area under the ROC curve** informs in a single numerical value about the overall diagnostic accuracy of the index test.

The **intended use** of a medical test can be diagnosis, screening, staging, monitoring, surveillance, prediction or prognosis. The **clinical role** of a test explains its position relative to existing tests in the clinical pathway. A replacement test, for example, replaces an existing test. A triage test is used before an existing test; an add-on test is used after an existing test.

Besides diagnostic accuracy, several other outcomes and statistics may be relevant in the evaluation of medical tests. Medical tests can also be used to classify patients for purposes other than diagnosis, such as staging or prognosis. The STARD list was not explicitly developed for these other outcomes, statistics, and study types, although most STARD items would still apply.

DEVELOPMENT

This STARD list was released in 2015. The 30 items were identified by an international expert group of methodologists, researchers, and editors. The guiding principle in the development of STARD was to select items that, when reported, would help readers to judge the potential for bias in the study, to appraise the applicability of the study findings and the validity of conclusions and recommendations. The list represents an update of the first version, which was published in 2003.

More information can be found on http://www.equator-network.org/reporting-guidelines/stard.

For peer review only - http://bmjopen.bmj.com/site/about/guidelines.xhtml
